# Network Pharmacology-Guided Evaluation of Ginger and Cornelian Cherry Extracts Against Depression and Metabolic Dysfunction in Estrogen-Deficient Chronic Stressed Rats

**DOI:** 10.3390/ijms26104829

**Published:** 2025-05-18

**Authors:** Nara Lee, Ting Zhang, Hanbin Joe, Sunmin Park

**Affiliations:** 1 Department of Food and Nutrition, Obesity/Diabetes Research Center, Hoseo University, Asan 31499, Republic of Korea; zzzz8238@naver.com; 2 Department of Bioconvergence, Hoseo University, Asan 31499, Republic of Korea; zhangting92925@gmail.com; 3 Department of Food Science & Technology, Hoseo University, Asan 31499, Republic of Korea; hanbinjoe@naver.com

**Keywords:** network pharmacology, memory deficit, HPA axis, estrogen deficiency, serotonergic synapse regulation

## Abstract

This study investigated the therapeutic effects of water extracts from *Zingiber officinale* Roscoe (ginger) and *Cornus officinalis* Siebold and Zucc. fruits (COF) water extracts on depression-like behavior and metabolic dysfunction in estrogen-deficient rats exposed to chronic mild stress (CMS). Network pharmacology analysis identified three bioactive compounds in ginger and four in COF, with 11 overlapping targets linked to both depression and metabolic pathways, primarily involving *NR3C1*, *HTR2A*, *MAOA*, and *SLC6A4* genes associated with hypothalamic–pituitary–adrenal (HPA) axis regulation and neurotransmitter modulation. Ovariectomized rats received 200 mg/kg/day of ginger or COF extracts for 7 weeks, with a 4-week CMS protocol initiated at week 3. Both extracts significantly improved depression-like behaviors, memory performance, glucose tolerance, lipid profiles, and bone mineral density, normalized HPA axis markers (corticosterone and ACTH), and increased hippocampal serotonin and dopamine levels. Ginger demonstrated greater efficacy in improving memory and metabolic outcomes compared to COF. Molecular docking further validated these findings, revealing strong and stable interactions between key phytochemicals—such as hydroxygenkwanin and telocinobufagin—and target proteins MAOA, HTR2A, and NR3C1, supporting their mechanistic role in stress and mood regulation. These results support supplementing ginger and COF extracts as promising botanical interventions for estrogen-deficiency-related mood and metabolic disorders, with potential clinical application at a human-equivalent dose of 1.5 g/day.

## 1. Introduction

Chronic psychological and physical stress, particularly in an estrogen-deficient state, is pivotal to the development of mood disorders, cognitive impairments, and systemic metabolic disturbances [1]. The hypothalamic–pituitary–adrenal (HPA) axis serves as a central mediator of these pathophysiological changes [2], with its sustained activation leading to glucocorticoid overproduction, hippocampal atrophy, serotonergic dysfunction, insulin resistance, and bone mineral loss [3,4]. Estrogen deficiency significantly exacerbates these effects by dysregulating the HPA axis, enhancing systemic inflammation, and impairing neurogenesis, which positions the menopausal transition as a critical window of vulnerability for comorbid depression and metabolic dysfunction [1,5].

Few animal models effectively capture their interconnected pathophysiology, particularly under conditions of estrogen deficiency. The combination of chronic mild stress (CMS) and ovariectomy (OVX) in animal models provides a clinically relevant approach that mimics the neuroendocrine, behavioral, and metabolic disturbances observed in postmenopausal women [6]. This estrogen-deficient stress model replicates key clinical features—including neuroendocrine disruption, behavioral despair, cognitive decline, insulin resistance, and bone demineralization—thereby providing a robust platform to explore interventions that address both mood and metabolic dysfunction in an integrated manner [6,7].

In recent years, herbal medicines have emerged as promising multi-target therapeutics for such complex conditions due to their diverse bioactive constituents and multifaceted mechanisms of action. Among them, *Zingiber officinale* Roscoe (ginger) and *Cornus officinalis* Siebold and Zucc. fruits (*Cornelian cherry*) have a long history in traditional medicine for alleviating fatigue, emotional instability, and metabolic irregularities. Ginger contains active compounds—primarily gingerols and shogaols—that modulate monoaminergic signaling, reduce neuroinflammation, and enhance glucose uptake in muscle and adipose tissues [8]. Complementarily, Cornelian cherry contains iridoid glycosides and loganin derivatives with documented antioxidant, anti-inflammatory, and insulin-sensitizing properties [9,10]. Notably, both botanicals affect central (mainly HPA axis-mediated) and peripheral (directly metabolic-mediated) mechanisms, making them strong candidates for addressing estrogen-deficiency-related syndromes [9,11].

The overlapping yet distinct bioactive profiles of ginger and Cornelian cherry suggest potential complementary or synergistic effects that have not been previously investigated in models of postmenopausal stress. Network pharmacology analysis of these herbs reveals interactions with key molecular targets including nuclear receptor subfamily 3 group C member 1 (*NR3C1*; glucocorticoid receptor), 5-hydroxytryptamine receptor 2A (*HTR2A*; serotonin receptor 2A), monoamine oxidase A (*MAOA*), and serotonin transporter gene (*SLC6A4*), which are critical regulators at the intersection of mood, stress response, and metabolic function [12]. These shared molecular targets provide a mechanistic rationale for investigating their combined effects on neuroendocrine regulation and metabolic function in estrogen-deficient states.

Previous research has demonstrated that ginger can modulate *HTR2A* and *MAOA* expression [8], while Cornelian cherry exhibits metabolic and anti-fatigue benefits [9,11]. However, no study has evaluated their combined therapeutic potential in addressing the comorbid neuroendocrine and metabolic disturbances characteristic of estrogen deficiency with chronic stress. Based on these converging lines of evidence, we hypothesized that water extracts of ginger and Cornelian cherry (COF) would ameliorate depression-like behavior and metabolic dysfunction in estrogen-deficient rats by regulating shared neuroendocrine and metabolic pathways, specifically via HPA axis normalization, neurotransmitter system modulation, and direct metabolic regulation. This study aims to (1) identify overlapping molecular targets of ginger and COF that mediate both mood and metabolic effects; (2) validate these therapeutic mechanisms in an OVX-CMS rat model; (3) determine whether their combined administration provides enhanced benefits compared to either extract alone; and (4) support these mechanistic insights with molecular docking analyses that highlight key ligand–receptor binding mechanisms between major bioactive compounds of ginger and COF with critical regulatory proteins including NR3C1, HTR2A, MAOA, and SLC6A4. This in silico approach enables the prediction of binding affinities and comparative evaluation of individual versus combined phytochemical interactions, providing structural insight into their multi-target therapeutic potential.

## 2. Results

### 2.1. Network Pharmacology-Based Analysis

**Figure 1:**
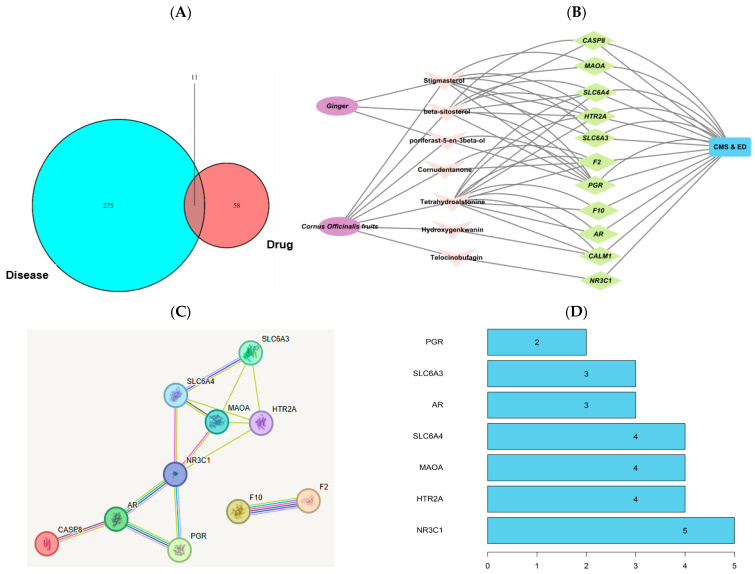
Network pharmacological analysis. (**A**) Venn diagram of the interaction between natural compounds and disease. (**B**) Herbs (ginger and Cornelian cherry water extracts (COF))—natural compounds—target genes—disease network diagram (natural components are marked in pink, disease gene targets in green). (**C**) String interaction of genes. Known interactions: Sky blue line, from curated databases; pink line, experimentally determined. Predicted interactions: green line, gene neighborhood; red line, gene fusions; blue line, gene co-occurrence. Others: light green line, text mining; black, co-expression; violet line, protein homology. (**D**) Protein–protein network analysis to identify the number of natural compounds in ginger and COF associated with the target genes. *NR3C1*: glucocorticoid receptor (nuclear receptor subfamily 3, group C, member 1), *HTR2A:* serotonin receptor 2A (5-hydroxytryptamine receptor 2A), *MAOA:* monoamine oxidase A, and *SLC6A4*: Solute Carrier Family 6 Member 4 (serotonin transporter).

Network pharmacology analysis identified 13 bioactive compounds in ginger and 20 in COF, based on selection criteria of oral bioavailability (OB) and drug-likeness (DL) scores (Table S1). Correspondingly, 52 target genes were associated with ginger and 70 with COF (Table S1). A total of 286 disease targets associated with CMS and estrogen deficiency were identified. There was an overlap of seven bioactive compounds and 11 disease targets linked with the two conditions (Figure 1A). The Cytoscape version 3.10.2 analysis revealed that three natural compounds from ginger interacted with these genes related to chronic stress and estrogen deficiency and four from COF also showed interactions (Figure 1B). These genes, except F2 and F10, were connected with NR3C1 (Figure 1C). The PPI network identified key genes with high connectivity, including *NR3C1*, *HTR2A*, *MAOA*, and *SLC6A4*, suggesting their pivotal roles in the stress and estrogen deficiency pathways (Figure 1D). The bioactive components of ginger, such as beta-sitosterol, stigmasterol, and poriferast-5-en-3beta-ol, primarily influenced these targets. Telocinobufagin and tetrahydroalstonine, unique to COF, also interacted with these genes.

### 2.2. Gene Ontology (GO) and Kyoto Encyclopedia of Genes and Genomes (KEGG) Enrichment Analyses

**Figure 2:**
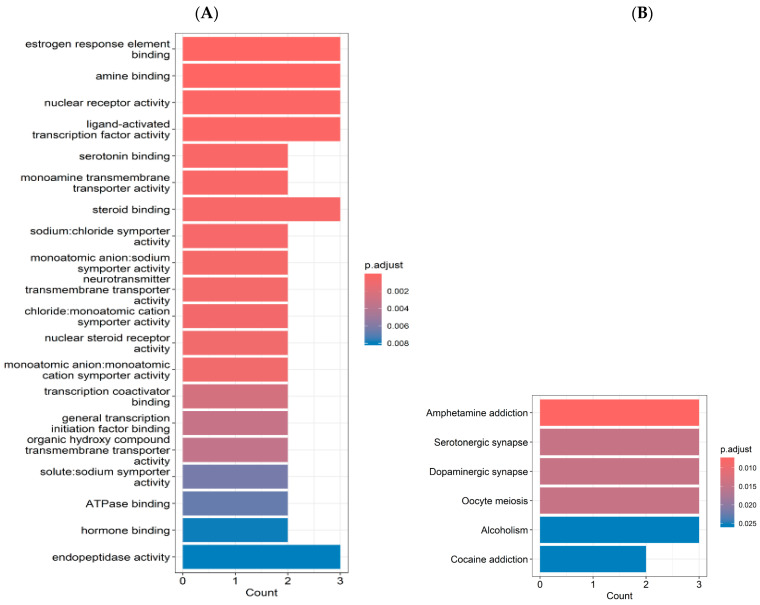
Involvement between chronic stress (CMS), estrogen deficiency, ginger, and Cornelian cherry water extracts (COF). (**A**) Gene ontology (GO) enrichment analysis (**B**) Kyoto Encyclopedia of Genes and Genomes (KEGG) enrichment analysis. Red boxes of the signal pathways represent the critical molecular targets for ginger and COF active compounds in CMS and estrogen deficiency. Analysis was conducted using KEGG from Kanehisa Laboratories, Kyoto University (Kyoto, Japan) (https://www.kegg.jp/, accessed on 18 November 2024).

GO enrichment analysis revealed a significant involvement of pathways related to estrogen response element binding, amine binding, nuclear receptor activity, ligand-activated transcription factor activity, and steroid binding (counts = 3.0; *p* < 0.002; Figure 2A). Additionally, serotonin binding, monoamine transmembrane transporter activity, and neurotransmitter transmembrane transporter activity were enriched (counts = 2.0; *p* < 0.002). The KEGG pathway analysis showed enrichment in the pathways related to brain function, including amphetamine addiction, serotonergic synapse, and dopaminergic synapse (counts = 3.0; *p* < 0.02; Figure 2B).

### 2.3. The Contents of Index Compounds in Ginger and COF

The amounts of index compounds in ginger and COF are presented in Table 1, and their chromatograms are provided in Figure S1. Ginger mainly contained 6-gingerol (6.25 mg/g) and 8-gingerol A and B (1.36 and 1.31 mg/g). COF contained morroniside (5.695 mg/g) and loganin (3.476 mg/g) (Table 1).

### 2.4. Energy and Glucose Metabolism

Serum 17β-estradiol concentration was lowered more in OVX than Sham rats by about 4.5-fold, and CMS decreased the levels by about 20% only in the Sham rats (Table 2). Ginger and COF intake marginally but significantly increased serum 17β-estradiol concentration. Uterine weight also showed a similar pattern with serum 17β-estradiol concentrations (Table 2). OVX increased body weight gain compared to Sham by about 25% regardless of CMS, while CMS decreased it with no significant effect of ginger or COF intake (*p* < 0.05). OVX significantly increased visceral fat mass compared to Sham (*p* < 0.01), while CMS reduced it (*p* < 0.01). Ginger but not COF lowered fat mass further than the OVX-CMS group (Table 2).

**Figure 3:**
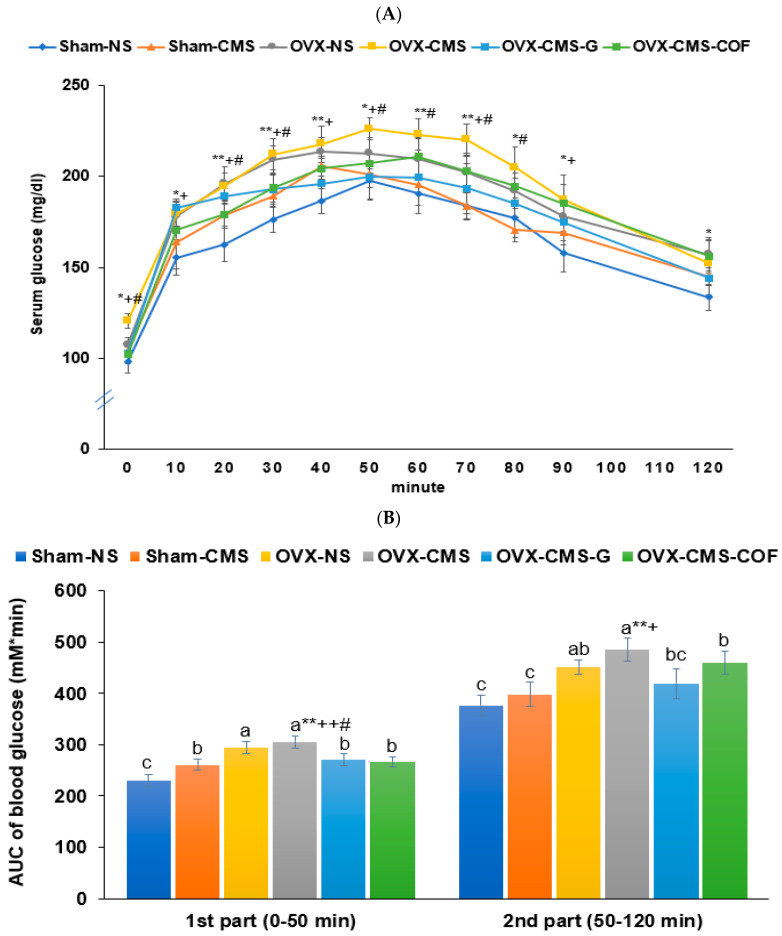
Changes in serum glucose concentrations (**A**) and area under the curve (AUC) of serum glucose levels (**B**) during oral glucose tolerance test following ginger or Cornelian cherry (COF) intervention in ovariectomized (OVX) rats under chronic mild stress (CMS). Sham-operated without CMS (Sham-No), sham-operated with CMS (Sham-CMS), OVX without CMS (OVX-No), OVX with CMS (OVX-CMS), OVX + CMS + ginger extract (OVX-CMS-G), and OVX + CMS + Cornelian cherry water extract (OVX-CMS-COF). * Significant main effect of OVX in two-way ANOVA at *p* < 0.05 and ** at *p* < 0.01. ^+^ Significant main effect of CMS in two-way ANOVA at *p* < 0.05 and ^++^ at *p* < 0.01. ^#^ Significant interaction between OVX and CMS in two-way ANOVA at *p* < 0.05. ^a,b,c^ Different superscript letters indicate significant differences between all six groups in the Tukey post hoc test at *p* < 0.05.

Fasting serum glucose and insulin levels were elevated in the OVX and CMS groups, while treatment with ginger and COF effectively reduced these elevations compared to OVX-CMS (*p* < 0.05; Table 2). Homeostasis model assessment for insulin resistance (HOMA-IR), representing insulin resistance, was highest in the OVX-CMS group and significantly improved with both treatments, with COF showing a similar effect to Sham-No (Table 2). The oral glucose tolerance test (OGTT) results revealed significant glucose intolerance in the OVX-CMS group, and treatments with ginger and COF reduced glucose intolerance comparable to the Sham-CMS, but not the Sham-No groups (Figure 3A). OVX and CMS elevated the area under the curve (AUC) of serum glucose concentration in the first and second parts of OGTT, and OVX-CMS showed the highest AUC in both parts among the groups. Administration of ginger and COF prevented the AUC increase, similar to Sham-No (*p* < 0.05; Figure 3B).

The liver damage indices, as indicated by serum alanine aminotransferase (ALT) and aspartate aminotransferase (AST) activities, were significantly higher in the OVX group compared to the Sham group, and the effect of chronic mild stress (CMS) was more pronounced in OVX rats. Administration of ginger and COF reduced serum AST activity more effectively than in the Sham-No group, and serum ALT levels in the OVX-CMS-G and OVX-CMS-COF groups were comparable to those of the Sham group (Table 2). These results suggest that daily intake of ginger and COF at 200 mg/kg bw/day did not induce liver toxicity and may even exert a protective effect under CMS conditions.

### 2.5. Impact of Metabolic and Inflammatory Biomarkers on Neurochemical Signaling in CMS

Serum triglycerides and total cholesterol were highest in the OVX-CMS group, while treatment with ginger and COF decreased these levels (*p* < 0.05; Table 3). Notably, treatment with ginger extract normalized triglycerides to the levels seen in the Sham-No group. HDL cholesterol, which was lowest in rats in the OVX-CMS group, improved with both treatments to levels seen in the Sham-CMS group (*p* < 0.01; Table 3). The inflammatory markers (tumor necrosis factor-alpha (TNF-α) and interleukin-beta (IL-1β)) were significantly elevated in the OVX-CMS group by about 24% but were reduced with the intake of ginger and COF (Table 3).

### 2.6. Bone Mineral Density (BMD)

**Figure 4:**
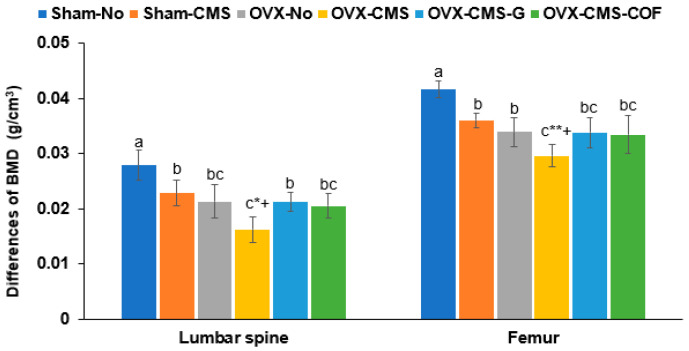
Bone mineral density (BMD) differences in the lumbar spine and femur before and after ginger or Cornelian cherry (COF) intervention in ovariectomized (OVX) rats under chronic mild stress (CMS). Sham-operated without CMS (Sham-No), sham-operated with CMS (Sham-CMS), OVX without CMS (OVX-No), OVX with CMS (OVX-CMS), OVX + CMS + ginger extract (OVX-CMS-G), and OVX + CMS + Cornelian cherry water extract (OVX-CMS-COF). * Significant main effect of OVX in two-way ANOVA at *p* < 0.05 and ** at *p* < 0.01. ^+^ Significant main effect of CMS in two-way ANOVA at *p* < 0.05. ^a,b,c^ Different superscript letters indicate significant differences between all six groups in the Tukey post hoc test at *p* < 0.05.

Both OVX and CMS significantly decreased BMD in the lumbar spine and femur. Intake of ginger and COF extracts effectively mitigated these reductions, restoring BMD to levels comparable to the OVX-No group, indicating their protective effects against CMS-induced bone loss (Figure 4).

### 2.7. Depression-like Behaviors by Sucrose Preference, Y-Maze, Forced Swimming, and Planar Tests

**Figure 5:**
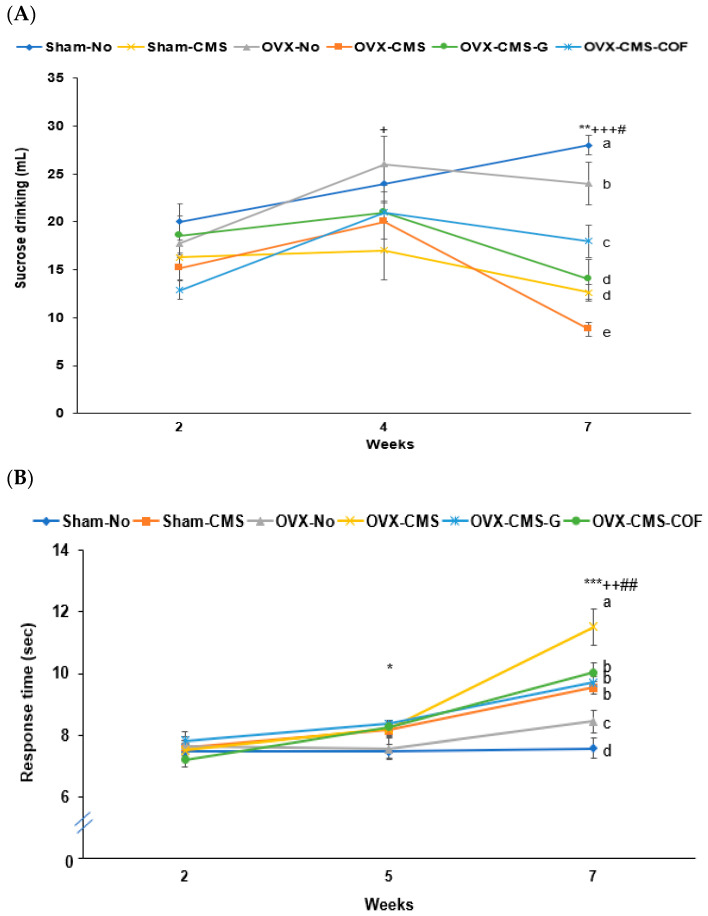

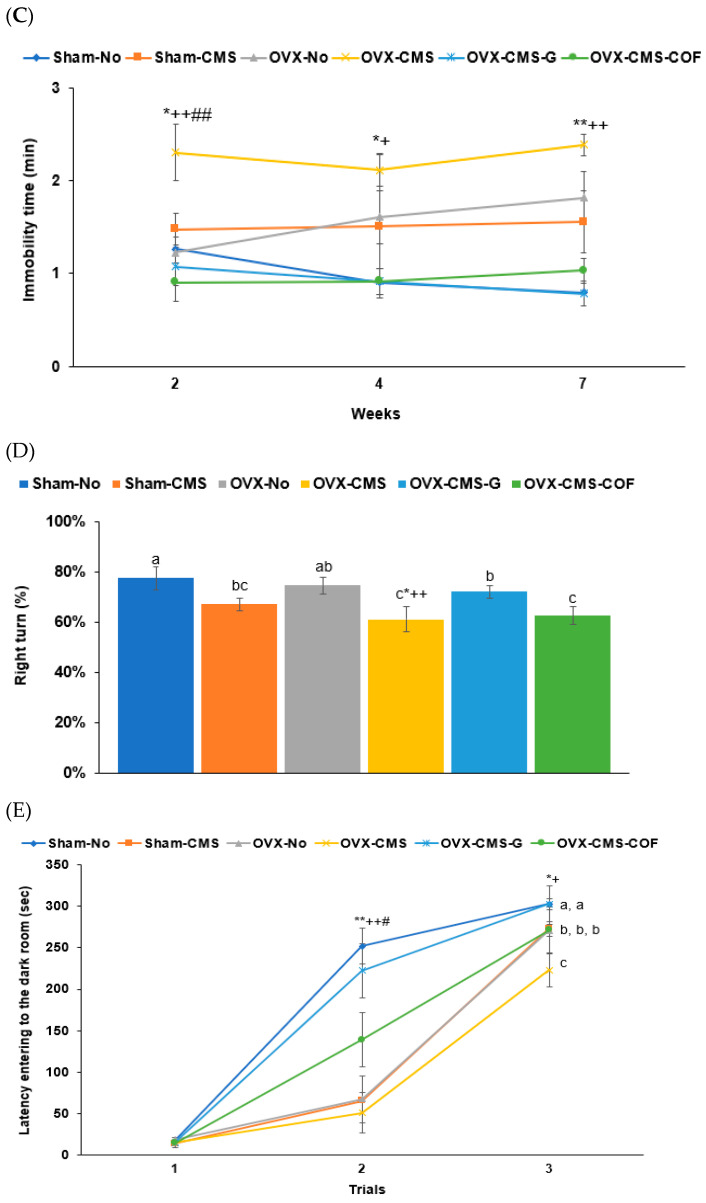

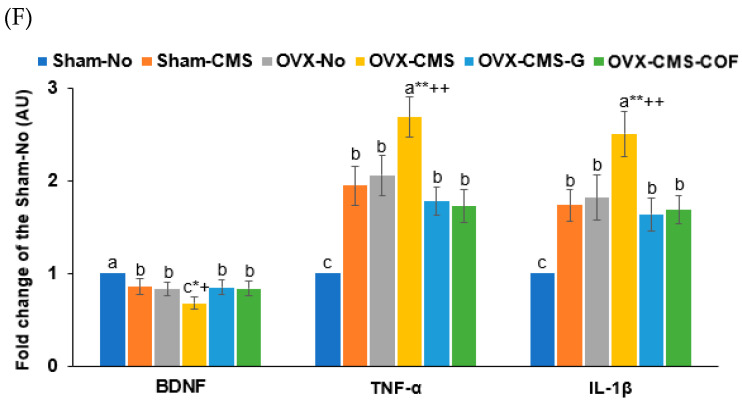
Behavioral performance and hippocampal mRNA expression following ginger or Cornelian cherry (COF) intervention in ovariectomized (OVX) rats under chronic mild stress CMS). (**A**) Sucrose solution drinking test. (**B**) Planar test. (**C**) Forced swimming test. (**D**) Y-maze test. (**E**) Passive avoidance test. (**F**) Relative mRNA measurement of hippocampal brain-derived neurotrophic factor (BDNF), tumor necrosis factor-alpha (TNF-α), and interleukin-1 beta (IL-1β). Sham-operated without CMS (Sham-No), sham-operated with CMS (Sham-CMS), OVX without CMS (OVX-No), OVX with CMS (OVX-CMS), OVX + CMS + ginger water extract (OVX-CMS-G), and OVX + CMS + Cornelian cherry water extract (OVX-CMS-COF). * Significant main effect of OVX in two-way ANOVA at *p* < 0.05, ** at *p* < 0.01, and *** at *p* < 0.001. ^+^ Significant main effect of CMS in two-way ANOVA at *p* < 0.05, ^++^ at *p* < 0.01, and ^+++^ at *p* < 0.001. ^#^ Significant interaction between OVX and CMS in two-way ANOVA at *p* < 0.05 and ^##^ at *p* < 0.01. ^a,b,c,d,e^ Different superscript letters indicate significant differences between all six groups in the Tukey post hoc test at *p* < 0.05.

Sucrose preference, reduced by CMS, was further decreased by OVX. Administration of ginger and COF partially restored sucrose preference by 1.7 and 2.3 folds, with COF showing a greater effect than ginger (Figure 5A). The planar test showed that CMS delayed the response time starting at week 5, and administration of ginger and COF prevented the delay. At week 7, OVX slightly increased response time, OVX-COF delayed the response time the most, and administration of ginger and COF decreased response time by about 28% as much as Sham-CMS (Figure 5B). This suggests that CMS delayed the response time the most, and OVX exacerbated the response time, and ginger and COF prevented the exacerbation. In forced swimming, CMS increased inactive time beginning at week 2 and continued to have a higher inactive time at 5 and 7 weeks (*p* < 0.05, *p* < 0.01), while OVX caused a more pronounced increment by week 5 and 7 compared to week 2 (Figure 5C). The OVX-CMS group exhibited the highest inactive times, which were significantly improved by treatment with ginger and COF by about 3.3- and 2.8-fold (Figure 5C). Ginger decreased the inactive time more than COF.

CMS affected memory function, which was assessed using the Y-maze and passive avoidance tests. In the Y-maze test, the right-turn percentage was reduced by both CMS and OVX treatments, but improved with ginger administration (Figure 5D). The passive avoidance test revealed increasing latency times across successive trials (Figure 5E). At the third trial, the OVX-CMS group showed the shortest latency to enter the dark chamber, while the Sham-No and OVX-CMS-G groups showed the longest latency times. OVX-CMS-COF, OVX-No, and Sham-CMS groups showed intermediate latency times (Figure 5E). Ginger intake in OVX and CMS rats improved memory function, equivalent to the Sham-No.

To evaluate the effects of the extracts on the HPA axis, the neurotransmitter levels were measured in various groups. CMS and OVX significantly increased serum corticosterone and adrenocorticotropic hormone (ACTH) concentrations by 1.5- and 2.6-fold, while reducing hippocampal serotonin levels by 3.5-fold (Table 3). Treatment with ginger and COF mitigated these changes, partly normalizing the HPA axis dysregulation and serotonin deficits associated with CMS and OVX. Hippocampal serotonin and dopamine contents were lower in the CMS and OVX rats, and treatment with ginger and COF increased the serotonin compared to the OVX-CMS, but not as much as that seen in the Sham-No rats (Table 3). Hippocampal serotonin and dopamine contents and their signaling were linked to *MAOA* and *HTR2A* genes, as shown in the network pharmacology analysis. Additionally, malondialdehyde (MDA) in the hippocampus also increased with OVX and CMS, and treatment with ginger and COF decreased the same. The increased MDA levels were linked to hippocampal superoxide dismutase (SOD) activity: the SOD activity was lower with OVX and CMS additively and treatment with ginger and COF extracts prevented a decrease in OVX and CMS (Table 3).

The relative abundance of brain-derived neurotrophic factor (*BDNF*) mRNA decreased with CMS and OVX, and they had a synergistic effect (Figure 5F). Treatment with ginger and COF increased it as much as in the OVX-No and Sham-CMS groups. *TNF-α* and *IL-1β* mRNA expressions increased with CMS and OVX, and treatment with ginger and COF decreased their expression as much as in the OVX-No group (Figure 5F).

### 2.8. Molecular Docking Analysis of Co-Crystallized Protein and Ligand (Redocking Validation)

To validate the reliability of the molecular docking protocol, redocking experiments were conducted using the co-crystallized ligands of their respective protein targets. This process involves re-docking the original ligand into the active site of the protein from which it was co-crystallized, to assess whether the docking algorithm can reproduce the known binding pose and interactions (Figure S2). For MAOA (PDB ID: 2BXR), the co-crystallized ligand clorgyline exhibited key interactions including amide–π stacking with Tyr-175, π–alkyl contacts with Leu-176 and Arg-172, alkyl interactions with His-187, and van der Waals forces with residues such as Glu-329, Glu-185, and Pro-186, resulting in a docking score of −5.0 kcal/mol (Figure S2A). Although clorgyline was co-crystallized, the moderate binding score reflects its relatively lower binding affinity in the docking simulation. In HTR2A (PDB ID: 6A93), the redocking of risperidone revealed predominantly hydrophobic interactions, such as π–alkyl contacts with Leu-325 and Ala-321, alkyl interactions with Lys-320, and van der Waals contacts with Asn-384, Asn-317, Val-324, Tyr-380, Leu-113, and Glu-318. Additionally, hydrogen bonds were observed with Asn-110 and Asn-107, culminating in a binding score of −8.5 kcal/mol (Figure S2B). For NR3C1 (PDB ID: 1NHZ), redocking of the co-crystallized ligand 11-(4-dimethylamino-phenyl)-17-hydroxy-13-methyl-17-prop-1-ynyl-dodecahydro-cyclopenta[a]phenanthrene-3-one revealed alkyl interactions with Tyr-478 and Val-449, π–alkyl interactions with Arg-479, and van der Waals contacts with Ser-708, Asn-454, and over 15 additional residues, with a final binding score of −7.1 kcal/mol (Figure S2C). These redocking results demonstrate favorable binding scores and the reproduction of critical native interactions, supporting the validity and robustness of the molecular docking methodology applied in subsequent studies of novel compounds.

### 2.9. Molecular Docking Analysis

**Figure 6:**
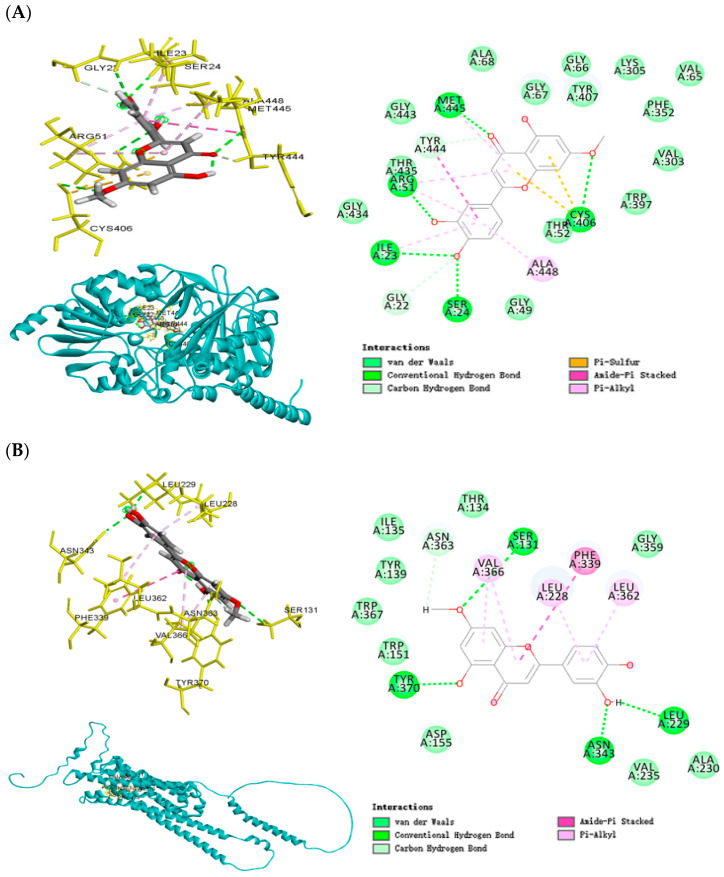

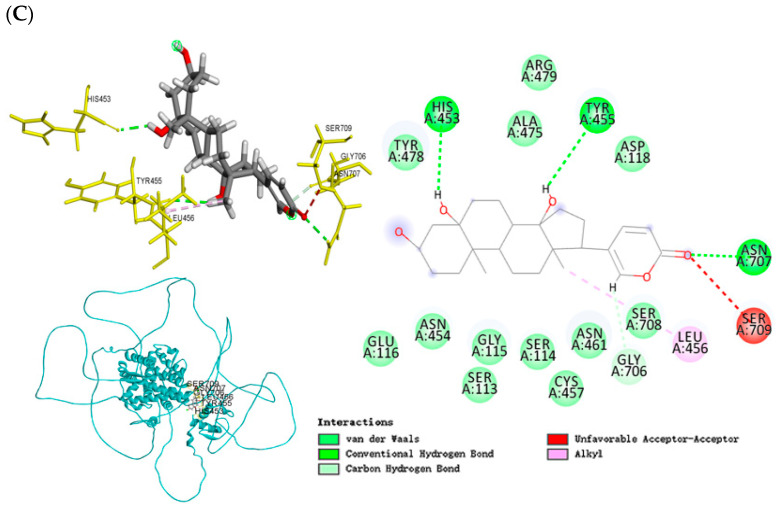
Molecular docking between proteins and selected natural compounds. (**A**) MAOA and hydroxygenkwanin (binding energy = −8.4). (**B**) HTR2A and hydroxygenkwanin (binding energy = −8.5). (**C**) NR3C1 and telocinobufagin (binding energy = −8.4).

This analysis provided structural insights into the interactions between key phytochemicals (hydroxygenkwanin and telocinobufagin) and target proteins, including MAOA, HTR2A, NR3C1, SLC6A3, and SLC6A4. Hydroxygenkwanin and telocinobufagin exhibited low binding energy values with the proteins (ranging from −8.2 to −8.5 kcal/mol), indicating strong binding affinities, and supported the predictions from the network pharmacology analysis (Figure 6, Table S2). Hydroxygenkwanin demonstrated strong binding to MAOA with a binding energy of −8.4 kcal/mol (Figure 6A). The ligand formed conventional hydrogen bonds with residues Met-445, Arg-51, Ile-23, Ser-24, and Cys-406, and also engaged in amide–π stacking and alkyl interactions with the Ala-448 residue, stabilizing its position deep within the catalytic pocket. The same compound, hydroxygenkwanin, also bound to the serotonin receptor 2A (HTR2A) with a binding energy of −8.5 kcal/mol (Figure 6B). Key stabilizing interactions included hydrogen bonding with Ser-131, Tyr-370, Leu-229, and Asn-343 residues, as well as π-alkyl interactions with Leu-228, Val-366, and Leu-362 residues, suggesting modulation of serotonergic signaling through receptor-level engagement. Telocinobufagin exhibited a strong binding affinity for the NR3C1 (−8.4 kcal/mol; Figure 6C). This compound formed multiple hydrogen bonds with His-453, Asn-707, and Tyr-455, and van der Waals contacts throughout the receptor’s ligand-binding domain, potentially affecting glucocorticoid signaling and stress hormone feedback regulation. These molecular docking simulations confirmed that major phytochemicals in ginger and COF can bind stably and specifically to key neuroendocrine and neurotransmitter targets, providing mechanistic support for their observed behavioral and metabolic effects in the OVX-CMS animal model. The interaction patterns—particularly involving HPA axis regulators (NR3C1) and monoamine-related proteins (MAOA, HTR2A)—strongly reinforce the multi-targeted therapeutic potential of these botanicals in estrogen-deficiency-associated stress conditions (Table S2).

## 3. Discussion

This study demonstrated that both ginger and COF treatments effectively alleviated CMS-induced depression-like behaviors and metabolic disturbances in OVX rats. These findings provide promising evidence for the use of natural bioactive compounds as non-hormonal therapeutic strategies for treating depression and metabolic dysfunction under estrogen-deficient conditions, which are particularly relevant to postmenopausal women facing chronic stress.

A key strength and novelty of this study lies in its integrated experimental design, which combines network pharmacology-based target prediction with in vivo validation in a dual pathology model encompassing both neuropsychiatric and metabolic disturbances. Unlike previous studies [13,14] that typically examine either mood or metabolic effects in isolation, our work uniquely explores how either ginger or COF supplementation impacts interconnected neuroendocrine pathways, including HPA axis regulation and monoaminergic neurotransmission, within the context of estrogen deficiency and CMS. Along with network pharmacology predictions, both ginger and COF improved depression-like symptoms and memory impairments by increasing serotonin and dopamine levels in the hippocampus through modulation of HPA axis activity. Notably, ginger demonstrated superior efficacy in addressing cognitive deficits compared to COF, supporting its potential role in preserving hippocampal function. These results indicate that while CMS exacerbates behavioral and metabolic symptoms associated with estrogen deficiency, supplementation with ginger or COF may mitigate these effects through multi-target mechanisms, supporting the case for future clinical evaluation.

Consistent with network pharmacology predictions, both extracts improved behavioral and cognitive impairments through modulation of the HPA axis, leading to increased serotonin and dopamine levels in the hippocampus. This aligns with earlier studies showing the importance of monoaminergic regulation in stress-induced depression [15]. Notably, ginger showed superior efficacy in memory enhancement, supporting prior evidence that gingerols and shogaols enhance synaptic plasticity and cognitive performance [16]. However, in the present study, gingerols and shogaols were excluded due to low levels of OB and DL and limited interactions with target genes for memory function. Their activities influenced the reduction in CMS. Importantly, the predicted involvement of steroid hormone signaling proved critical in our OVX-CMS model. Previous studies have shown that estrogen deficiency sensitizes the brain to stress, in part due to reduced expression of BDNF and compromised hippocampal neuroplasticity [16]. Our results confirmed that OVX-CMS rats had more pronounced behavioral and neuroendocrine disruptions, supporting the idea that estrogen withdrawal exacerbates the stress response, in line with clinical observations in postmenopausal women [17].

Estrogen deficiency appears to prime the brain for enhanced stress sensitivity, which is consistent with previous studies that postmenopausal women show greater susceptibility to stress-related disorders [1,18]. The loss of estrogen’s neuroprotective effects compromises the brain’s capacity to cope with CMS. Additionally, estrogen promotes *BDNF* mRNA expression and maintains synaptic plasticity in key brain regions [19]. Our findings that OVX rats exposed to CMS showed more severe behavioral impairments than either OVX or CMS-exposed rats, though the study has reported similar underlying mechanisms focusing on GABAergic modulation rather than our identified serotonergic and dopaminergic pathways [20,21]. Furthermore, some studies [21] have no additive stress effect in OVX rats with CMS, unlike our findings.

The interaction between estrogen deficiency and stress response centers around HPA axis regulation, where estrogen receptors modulate glucocorticoid receptor expression [1]. Our results showed that OVX rats had elevated baseline corticosterone levels, indicating impairment of the HPA axis negative feedback loop, similar to other findings [22,23]. When subjected to CMS, these rats exhibited an exaggerated stress response, with significantly higher corticosterone and ACTH levels than sham-operated CMS rats. This dysregulation aligns with a proposed model of chronic stress, further suppressing estrogen receptor signaling in brain regions already compromised by estrogen deficiency [24]. Notably, treatment with both ginger and COF effectively attenuated this HPA axis hyperactivation, as evidenced by normalized corticosterone and ACTH levels in treated OVX-CMS rats. This improvement in HPA axis function likely contributed to the observed reduction in depression-like behavior, supporting the conclusion that natural compounds can restore neuroendocrine balance under both estrogen deficiency and CMS conditions [25].

The behavioral improvements observed in our study strongly aligned with the molecular mechanisms predicted by network pharmacology analysis. Treatment with ginger and COF significantly improved performance across multiple behavioral tests, including the Y-maze, forced swimming, and planar tests, suggesting comprehensive effects on both cognitive and emotional behaviors. Some studies consistently reported improved spatial memory with natural compounds targeting monoamine pathways [26,27]. The enhanced Y-maze performance, indicating improved spatial memory and cognitive function, can be attributed to the regulation of both serotonergic and dopaminergic synapses, as identified by previous studies [26,28]. Network pharmacology identified compounds in ginger and COF that target key monoamine transporters, including the serotonin transporter and dopamine transporters, which is consistent with other findings on natural compounds and neurotransmitter modulation [29,30].

The metabolic disturbances in OVX-CMS rats, including elevated HOMA-IR and glucose intolerance, are consistent with clinical data linking estrogen loss and CMS to insulin resistance [31,32]. Elevated corticosterone levels, as seen in our model, are known to impair insulin signaling, validating our model and mechanistic approach [33]. Both treatments reduced HOMA-IR and improved glucose tolerance, suggesting that stress-metabolic coupling can be therapeutically targeted through natural product intervention, in agreement with results from other studies examining anti-diabetic effects of ginger and Crataegus species [31,34]. Improved insulin sensitivity was further supported by favorable lipid profiles and glucose handling, reinforcing the finding that attenuation of HPA hyperactivation mediates not just neuroendocrine, but also metabolic restoration. These findings are aligned with integrative models of stress-induced metabolic dysfunction, where glucocorticoid overload is a central mechanism driving insulin resistance and dyslipidemia [35].

Our molecular docking analysis provides significant mechanistic insight into how bioactive compounds from ginger and COF may affect neuroendocrine and neurotransmitter systems implicated in stress responses. Through computational analysis, we identified key phytochemicals—particularly hydroxygenkwanin and telocinobufagin—that demonstrated remarkably strong binding affinities to major stress-related targets (MAOA, HTR2A, and NR3C1), with docking energies ranging from −8.4 to −8.5 kcal/mol. These favorable binding energies suggest potential biological activity that could influence critical pathways involved in mood regulation and stress response. Importantly, while computational docking scores provide valuable predictive insights, they represent approximations of binding free energy rather than direct measurements of biological activity. Generally, binding energies below −8.0 kcal/mol indicate strong potential interactions, with lower values correlating with higher binding affinities and potentially greater biological effects in vivo [36]. However, the translation from computational binding energy to actual biochemical activity depends on additional factors, including bioavailability, metabolic stability, and protein conformational dynamics that cannot be fully captured in rigid docking simulations. Their biological activity needs to be confirmed with in vitro or in vivo experiments.

The validation of our docking protocol using co-crystallized ligands as reference standards strengthens the reliability of our findings. Redocking experiments demonstrated robust performance, reproducing critical native interactions with binding scores ranging from −5.0 to −8.5 kcal/mol for co-crystallized ligands such as clorgyline (MAOA), risperidone (HTR2A), and the NR3C1 ligand. Notably, hydroxygenkwanin’s binding energy with HTR2A (−8.5 kcal/mol) matched that of risperidone, a clinically effective antipsychotic with known serotonergic activity, suggesting comparable target engagement potential. Hydroxygenkwanin’s affinity for MAOA (−8.4 kcal/mol) significantly exceeded clorgyline’s binding energy (−5.0 kcal/mol), indicating potentially superior binding characteristics. This binding profile shares characteristics with established MAOA inhibitors [37], suggesting a potential mechanism for influencing central monoamine turnover that could explain the observed behavioral effects. Similarly, for HTR2A, hydroxygenkwanin’s interactions with residues are functionally relevant for receptor conformation and activation of parallel binding sites utilized by serotonergic compounds [38], suggesting potential modulation of serotonergic neurotransmission through either direct agonism or allosteric mechanisms. Telocinobufagin demonstrated enhanced binding to NR3C1 (−8.4 kcal/mol) compared to its co-crystallized ligand (−7.1 kcal/mol), with its interaction with critical residues for glucocorticoid binding indicating a possible role in HPA axis regulation through alternative binding modes that may either mimic or modulate endogenous corticosteroid activity [39]. These comparative binding profiles, characterized by extensive hydrogen bonding and π-alkyl interactions, provide compelling mechanistic support for the multi-targeted therapeutic potential of ginger and COF phytochemicals in modulating both HPA axis regulation and monoamine signaling pathways in estrogen-deficiency-associated stress conditions, though future biochemical and cellular assays will be essential to confirm the predicted activities in physiologically relevant systems.

Although COF-derived compounds such as telocinobufagin exhibited strong docking affinities, the ginger-treated group displayed more robust behavioral improvements in the OVX-CMS model. This apparent disconnect underscores the multifactorial nature of botanical pharmacology, where therapeutic outcomes depend not only on receptor binding but also on bioavailability, metabolic effects, and synergistic interactions among phytochemicals. One possible explanation is the superior metabolic regulatory effects of ginger, including enhancements in glucose homeostasis, insulin sensitivity, and lipid metabolism [40], which may contribute indirectly to stress resilience and cognitive function. This hypothesis is supported by previous research showing that metabolic health is closely linked to HPA axis stability and mood regulation, particularly in postmenopausal populations [5]. Moreover, the primary active components in ginger—gingerols and shogaols—are known to cross the blood–brain barrier and modulate central neurotransmission and neuroinflammation, potentially amplifying behavioral effects, even when binding affinities are comparatively lower [41,42]. Together, these findings highlight the importance of integrating computational, behavioral, and metabolic data in evaluating the therapeutic potential of multi-target botanical interventions. This integrative perspective strengthens the translational relevance of ginger and COF as complementary candidates for treating stress-related menopausal dysfunction.

### Limitations and Future Directions

While our results are promising, several limitations must be acknowledged. First, we used sham-operated controls with and without CMS instead of a positive control, such as estrogen replacement or standard antidepressants, which would have provided direct comparisons to current clinical treatments. Second, we evaluated only a single dose of ginger and COF (200 mg/kg/day), limiting our understanding of dose-dependent effects. A comprehensive dose–response study would better inform potential clinical applications. Additionally, while our study examined ginger and COF separately, we did not investigate their potential synergistic effects when administered together. Given their complementary mechanisms identified through network pharmacology, future studies should explore combined administration to determine if enhanced therapeutic effects can be achieved at lower doses.

Further mechanistic studies are needed to fully elucidate the molecular pathways through which these extracts exert their effects. While our network pharmacology provided valuable predictions, direct experimental validation of target engagement and downstream signaling would strengthen the mechanistic understanding. Finally, our findings in an animal model, while promising, require validation in human clinical trials. The translation of these findings to postmenopausal women experiencing stress-related depression and metabolic dysfunction represents an important next step in determining the clinical utility of ginger and COF as therapeutic interventions.

## 4. Materials and Methods

### 4.1. Network Pharmacology-Based Analysis

The active ingredients of ginger and COF were identified using the traditional Chinese medicine systems pharmacology database and analysis platform (TCMSP; https://tcmsp-e.com/tcmsp.php; accessed on 11 October 2024) and the Encyclopedia of Traditional Chinese Medicine (ETCM) (http://www.tcmip.cn/ETCM/index.php/Home/; accessed on 15 October 2024). Screening criteria included OB ≥ 30% and DL ≥ 0.15 since the index compounds of ginger, 6-gingerol, and 6-shogaol had scores of 0.16 and 0.15, respectively. Potential targets of these active ingredients were retrieved from the TCMSP database [43]. The GeneCards^®^ (Rehovot, Israel) database (https://www.genecards.org/; accessed on 17 October 2024) was utilized to identify targets related to CMS and estrogen deficiency, and a relevance score threshold of >20 was applied. Additional targets were obtained from the online Mendelian inheritance in man database (https://www.omim.org/; accessed on 22 October 2024) using keywords such as “chronic mild stress”, “stress”, and “estrogen deficiency”. Overlapping genes associated with chronic stress, estrogen deficiency, ginger, and COF were identified and visualized through a Venn diagram to pinpoint potential therapeutic targets.

The bioactive compounds identified through the screening and their intersecting targets were imported into Cytoscape 3.9.1 to construct a compound-target network. Network topology analysis was conducted using the “analyze network” tool within Cytoscape [43]. The intersecting targets were further analyzed using the search tool for the retrieval of interacting genes/proteins database (https://string-db.org/; accessed on 27 October 2024), selecting “*Homo sapiens*” as the species to construct a protein–protein interaction (PPI) network. Functional and pathway enrichment analyses were performed to explore the biological significance of the intersecting targets.

Pathway analysis was conducted using the KEGG database, which provides comprehensive information on molecular interactions and biochemical pathways. Functional analysis was carried out in GO terms, focusing on biological processes, cellular components, and molecular functions. The Bioconductor version 3.9 open-source software for the bioinformatics platform was employed to facilitate high-throughput data interpretation.

### 4.2. Molecular Docking Analysis

Molecular docking was conducted to explore the interactions between bioactive compounds from ginger and Cornelian cherry with key target proteins identified through network pharmacology. The amino acid sequences of selected target proteins—NR3C1 (glucocorticoid receptor), HTR2A (serotonin receptor 2A), MAOA (monoamine oxidase A), and SLC6A4 (serotonin transporter)—were retrieved from the UniProt database. For each target protein, the corresponding 3D structure was retrieved from the Protein Data Bank (PDB) and verified to ensure the protein sequence matched the structure. In cases where no suitable PDB structure was available, high-resolution 3D models were generated using the amino acid sequences through the SWISS-MODEL homology modeling server (https://swissmodel.expasy.org/; accessed on 6 December 2024). The PDB IDs used for molecular docking analysis were MAOA (2BXR), HTR2A (6A93), and NR3C1 (1NHZ), which were selected based on optimal crystallographic resolution and refinement [44]. If the protein is not a monomer, the monomer was extracted using Discovery Studio. Each retrieved protein structure contained co-crystallized ligands representing known agonists or antagonists of their respective receptors. The co-crystalized ligands for MAOA (2BXR), HTR2A (6A93), and NR3C1 (1NHZ), were flavin-adenine dinucleotide, risperidone, and 11-(4-dimethylamino-phenyl)-17-hydroxy-13-methyl-17-prop-1-ynyl-1,2,6,7,8,11,12,13,14,15,16,17-dodec-anhydro-cyclopenta[A]phena nthren-3-one, respectively. These ligands were removed during preprocessing to eliminate potential bias and ensure unoccupied binding sites for unbiased docking of the selected phytochemicals. Protein structures were prepared using Discovery Studio, where water molecules and co-crystallized ligands were removed, and structural anomalies were corrected. Protonation and assignment of Kollman charges were performed using AutoDock Tools v1.5.6, and the protein structures were converted into PDBQT format for compatibility with the docking workflow. To validate the molecular docking protocol, the co-crystallized ligands were re-docked into their corresponding protein binding sites.

Bioactive compounds selected for docking included major phytochemical constituents of ginger and COF, based on prior phytochemical analyses. These included 6-gingerol, 6-shogaol, loganin, morroniside, hydroxygenkwanin, and telocinobufagin, which were identified through compound-target screening. The chemical structures of these compounds were retrieved from PubChem (https://pubchem.ncbi.nlm.nih.gov; accessed on 13 December 2024) using the following CID numbers: 6-shogaol, 5281794; loganin, 87691; morroniside, 11228693; hydroxygenkwanin, 5318214; and telocinobufagin, 259991. The 2D structures were converted to 3D conformers and energy-minimized using Open Babel. All ligands were then converted to PDB format and subsequently to PDBQT format using AutoDock Tools to enable flexible molecular docking. During preprocessing, rotatable bonds were defined and appropriate Kollman charges were assigned.

Active site prediction was performed using the ProteinsPlus web server, focusing on conserved and functional pockets. AutoDock Vina v1.2 was used for docking, with a grid box defined around the predicted binding site [45]. Docking simulations applied an exhaustiveness level of 8–12 for accuracy. Ligands were evaluated based on binding affinities, with values lower than −8.0 kcal/mol considered significant. Top binding poses were analyzed using Discovery Studio, with emphasis on hydrogen bonds, hydrophobic interactions, and key contact residues. This integrated workflow ensured the reliable prediction of potential ligand–target interactions.

### 4.3. Preparation of Ginger and COF Water Extracts and Quantification of Index Compounds

Dried ginger root and COF were acquired from the Kyung Dong Herbal Market (Seoul, Republic of Korea) and stored in the Korean Institute of Oriental Medicine, confirmed by Dr. Ko BS. Each sample was extracted in distilled water (1:10, *w*/*v*) at 80 °C for 24 h with continuous shaking. After filtration, the aqueous extracts were concentrated under reduced pressure using a vacuum rotary evaporator (Büchi, Flawil, Switzerland) at 60 °C to remove water while preserving thermolabile constituents. The concentrated extracts were subsequently lyophilized (Beckman Coulter, Indianapolis, IN, USA), yielding dry extract powders with extraction yields of approximately 16.8% for ginger and 11.4% for COF, calculated based on the original dry weight. Lyophilized extracts were stored at −20 °C until use.

Each dried ginger and COF water extract was dissolved in 100% methanol (10 mg/mL), filtered through a 0.45 μm PTFE syringe filter, and subjected to HPLC analysis using a YL-9100 system (Youngin Instrument, Seoul, Republic of Korea), equipped with a YL9120 UV/Vis Detector, YL9110 quaternary solvent delivery pump, YL9101 vacuum degasser, YL9130 column compartment, and a 7725i manual injector. Chromatographic separation was performed on a SunFire C18 column (100 Å, 5 μm, 4.6 × 250 mm; Waters, Milford, MA, USA). For ginger component analysis (6-gingerol, 8-gingerol A and B, 6-gingerdiol, 6-gingerdione, 10-gingerol, and 8-gingerdione), gradient elution was employed with solvent A (water with 0.1% formic acid) and solvent B (methanol), under the following conditions: flow rate of 1.0 mL/min, column temperature 30 °C, detection wavelength 280 nm, injection volume 10 μL, and total run time of 30 min. For COF analysis, morroniside and loganin were analyzed using isocratic elution with a mobile phase composed of water, methanol, and acetic acid (75:25:0.2, *v*/*v*). The flow rate was 0.9 mL/min, the column temperature was 30 °C, the detection wavelength was 240 nm, and the injection volume was 10 μL. The run time was 60 min. Calibration curves for all standard compounds were constructed using six concentrations ranging from 0.1 to 200 µg/mL, demonstrating excellent linearity with correlation coefficients (R^2^) exceeding 0.999. The limits of detection (LOD) and limits of quantification (LOQ) were estimated based on signal-to-noise ratios of 3:1 and 10:1, respectively. Although precision at the lowest concentration points was limited, approximate LOD and LOQ values were determined as follows: 6-gingerol, LOD: 0.273 µg/mL, LOQ: 0.817 µg/mL; 8-gingerol, 0.176, 0.674 µg/mL; 10-gingerol, 0.052, 0.093 µg/mL; morroniside, 0.267, 0.810 µg/mL; and loganin, 0.215, 0.561 µg/mL. Despite potential underestimation at the lowest detection levels, the actual concentrations of all index compounds in the extract samples—ranging from 0.03 to 6.25 mg/g—were substantially higher than their respective LOD and LOQ thresholds. These results confirm that the quantification was both accurate and reproducible under the applied HPLC conditions. The number of bioactive compounds in each dried ginger or COF water extract (mg/g) was calculated by multiplying the concentration of bioactive compounds in the methanol solution (μg/mL), measured by HPLC, by the volume of methanol used to dissolve each extract (mL). Matrix effects were evaluated by comparing the signals of standard compounds spiked into the matrix (ginger and COF extracts) with those dissolved in methanol. No significant interference was observed, confirming that the HPLC analysis provided reliable quantification of the bioactive compounds in the extracts. Data were analyzed using the integrated chromatography software provided by the HPLC system (YL-9100).

### 4.4. Animals and Experimental Design

This study was conducted in compliance with the Guide for the Care and Use of Laboratory Animals (8th edition, National Institutes of Health) and was approved by the Institutional Animal Care and Use Committee of Hoseo University (HSIACUC-17-071). Female Sprague-Dawley rats (8 weeks old, 216 ± 13 g) were obtained from Daehan Bio Inc. (Eum-Sung, Republic of Korea) and maintained under controlled conditions (23 °C, 12-h light/dark cycle). After acclimatization, the rats underwent either bilateral ovariectomy (*n* = 50) or sham surgery (*n* = 20) under ketamine/xylazine anesthesia following previously described procedures [46]. The rats were randomly divided into six groups (*n* = 10 per group): sham-operated without CMS (Sham-No), sham-operated with CMS (Sham-CMS), OVX without CMS (OVX-No), OVX with CMS (OVX-CMS), OVX with CMS treated with ginger water extract (OVX-CMS-G), and OVX with CMS treated with COF water extract (OVX-CMS-COF). The dose of 200 mg/kg/day for both ginger and COF was selected based on previous studies demonstrating their efficacy in pain management and menopausal symptom relief, respectively, in both rodent models and human-equivalent doses in clinical research [34,47,48,49]. Ginger is classified as “Generally Recognized as Safe” (GRAS), and no safety concerns have been reported in the literature for COF at this dose [50]. The ginger and COF (200 mg/kg body weight/day) were administered orally using a Zonde each morning, and control groups for dextrin. On the day of the behavioral or physiological assessments, the extracts were given one hour before the assessment.

All the rats were fed a semi-purified diet containing 37% carbohydrates (corn starch and sucrose), 20% protein (casein), and 43% lard (CJ Co., Seoul, Republic of Korea), supplemented with vitamins and minerals according to the American Institute of Nutrition 98 diet, with unrestricted access to food and water throughout the study.

### 4.5. CMS Induction Protocol

The CMS protocol was adapted from the method established by Willner et al. [51] and was implemented over five weeks. The protocol involved a variety of mild stressors—including restraint, forced swimming, overnight illumination, tilted cages, and damp bedding—applied in a randomized and unpredictable sequence (Table S3). To maintain unpredictability and prevent habituation, the combination and order of stressors were varied each week using a computer-generated randomization schedule. The first two weeks were dedicated to stress induction only, followed by three weeks of concurrent stress and treatment exposure. Ginger and COF treatments were initiated three weeks prior to the start of CMS to model preventive dietary interventions, simulating real-world use of functional foods aimed at reducing the risk or severity of stress-related symptoms in postmenopausal individuals. This staggered design helped ensure that animals did not habituate to the stressors over time, preserving the model’s validity. Depression-like behaviors were assessed using multiple validated behavioral tests, including the sucrose preference test, forced swim test, and plantar test.

### 4.6. Behavioral Assessments

Depression-like symptoms were evaluated using the sucrose solution test, planar test, and Y-maze test following a standardized protocol [52]. The sucrose preference test, to measure anhedonia, was conducted according to the method described previously [53]. Rats were given access to two bottles, one containing water and the other containing a 2% sucrose solution, for 24 h. The percentage of sucrose solution consumed relative to total fluid intake was calculated. A lower preference for sucrose was indicative of anhedonia. The plantar test (Hargreaves method) was used to assess thermal nociception and pain sensitivity [54]. Each rat was individually placed in a transparent testing chamber and allowed to acclimate for 15 min. Using a plantar analgesia meter (IITC, Life Sciences Series 8 model 390; Woodland Hills, CA, USA), a focused beam of radiant heat was directed to the plantar surface of the rat’s hind paw. The latency time (in seconds) until the rat withdrew its paw from the heat source was recorded. Two measurements were taken for each rat to minimize error, and the average value was calculated for analysis. Longer withdrawal latency times indicated decreased pain sensitivity, which is typically associated with higher stress levels in chronic stress models.

Memory impairment was assessed with passive avoidance and a Y-maze test. In the passive avoidance apparatus with a two-compartment dark/light shuttle box, rats naturally enter the dark box when placed in the light shuttle box [22]. Electric stimulation (75 V, 0.2 mA, 50 Hz) was delivered to rats when they entered the dark box during two acquisition trials separated by 8 h. Sixteen hours after the second trial, the latency to enter the dark chamber was reassessed without electric stimulation to the foot. Latency was measured up to 600 s, with longer latency times indicating better memory function. The Y-maze test evaluated spatial memory and cognitive function in a Y-shaped maze. Correct alternations, defined as sequential entries into all three arms without repetition, were recorded and expressed as a percentage of total arm entries.

### 4.7. Glucose and Insulin Tolerance Tests

An OGTT was conducted after overnight fasting. Glucose (2 g/kg body weight) was administered orally, and serum glucose concentrations were measured at intervals (0, 10, 30, 60, 90, and 120 min) using a glucometer (Accu-Chek, Roche Diagnostics; Basel, Switzerland). On the following day, an intraperitoneal insulin tolerance test (IPITT) was performed after 6 h of fasting. Rats were administered 1 U/kg insulin intraperitoneally, and serum glucose concentrations were measured at similar intervals. The AUC for glucose was calculated for both tests. HOMA-IR was determined using the following formula: [serum insulin (μU/mL) × serum glucose (mmol/L)]/22.5.

### 4.8. BMD Measurement

BMD was measured at the beginning and the end of the experiment after the rats were anesthetized. BMD was measured in the legs and lumbar spine using dual-energy X-ray absorptiometry (Norland pDEXA Sabre) [55]. Differences in BMD were calculated to assess changes over the intervention period.

### 4.9. Tissue Collection and Biochemical Assays

At the end of the intervention, the rats were fasted for 16 h and anesthetized. Blood samples were collected from the portal vein and inferior vena cava. Epididymal and retroperitoneal fat masses were weighed to evaluate adipose tissue. Six brains per group were assigned for biochemical assays. The brains of four rats were fixed in 4% paraformaldehyde for histological studies. The hippocampus of each of the six brains was carefully dissected and divided into portions for biochemical assays and gene expression studies, and frozen at −80 °C. The hippocampal tissues for biochemical assays were homogenized in ice-cold RIPA lysis buffer (Thermo Scientific, Waltham, MA, USA) supplemented with protease and phosphatase inhibitor cocktail (Roche, Basel, Switzerland). Tissues were homogenized using a motorized tissue grinder and incubated on ice for 30 min with intermittent vortexing. Lysates were centrifuged at 14,000× *g* for 20 min at 4 °C to remove cellular debris. The supernatants were collected as total protein lysates and stored at −80 °C until further analysis.

Oxidative stress was assessed by measuring hippocampal MDA and SOD levels using a lipid peroxidation assay kit (Abcam, Cambridge, UK). To evaluate inflammation, the serum and brain levels of TNF-α and IL-1β were measured using an enzyme-linked immunoassay (ELISA) kit (eBioscience, San Diego, CA, USA). Serum ALT and AST activities were measured using Asan Pharmaceutical Inc. (Daejeon, Republic of Korea) in a Spectrophotometer. Serum corticosterone and adrenocorticotropic hormone levels, biomarkers of CMS, were analyzed using an ELISA kit. The hippocampal serotonin levels were evaluated using an ELISA kit (eBioscience).

RNA was isolated using a TRIzol reagent (Life Technologies, Rockville, MD, USA) according to the manufacturer’s protocol. For the cDNA synthesis, equal volumes of isolated RNA, Superscript III reverse transcriptase, and high-fidelity Taq DNA polymerase were combined. Gene expression was analyzed using SYBR Green-based real-time polymerase chain reaction (BioRad Laboratories, Hercules, CA, USA). Previously validated primers [56] were used to measure mRNA expression levels of neurotropic factors (*BDNF*) and inflammatory markers (*TNF-α* and *IL-1β*), with *β-actin* serving as the reference gene. Relative gene expression was calculated using the comparative threshold cycle (ΔΔCt) method for relative quantification [57].

### 4.10. Statistical Analysis

Statistical analysis was performed using SPSS version 22.0. Data were expressed as mean ± standard deviation (SD). A two-way analysis of variance (ANOVA) was performed to assess the main effects and interaction between OVX and CMS treatments across the Sham-No, Sham-CMS, OVX-No, and OVX-CMS groups. To evaluate the effects of ginger and COF, a one-way ANOVA was performed across all six groups (Sham-No, Sham-CMS, OVX-No, OVX-CMS, OVX-CMS-G, and OVX-CMS-COF), followed by Tukey’s post hoc test. A *p*-value of < 0.05 was considered statistically significant.

## 5. Conclusions

This study introduces a novel dual-herb therapeutic strategy using ginger and COF to target the interconnected neuroendocrine and metabolic dysfunctions associated with estrogen deficiency and CMS. Through an integrative approach combining network pharmacology, molecular docking, and in vivo validation, we demonstrated that these herbs significantly improved depression-like behavior, metabolic parameters, and HPA axis regulation in an OVX-CMS rat model. Molecular docking analysis supported the interaction of key phytochemicals with target proteins involved in glucocorticoid signaling, serotonin pathways, and oxidative stress, providing structural insight into their multi-target mechanisms. Ginger showed a stronger effect on cognitive impairment than COF, suggesting potential for targeted application. Together, these findings support the potential of ginger and COF, at a human equivalent dose of 1.5 g/day, as multi-target botanical therapeutics for managing stress-related disorders in postmenopausal women. Further clinical studies are warranted to confirm these benefits and explore translational applications.

## Figures and Tables

**Table 1:** The contents of index compounds in ginger and Cornelian cherry water extracts.

	Retention Time	Ginger Water Extract	Cornelian Cherry Water Extract
6-gingerol (mg/g)	6.502	6.25	
8-gingerol A (mg/g)	11.780	1.36	
8-gingerol B (mg/g)	18.507	1.31	
6-gingerdiol (mg/g)	20.557	0.09	
6-gingerdione (mg/g)	21.270	0.40	
10-gingerol (mg/g)	27.578	0.03	
8-gingerdione (mg/g)	41.705	0.61	
Morroniside (mg/g)	9.523		5.695
Loganin (mg/g)	23.258		3.476

**Table 2:** Estrogen status, adiposity, energy balance, glucose metabolism, and liver damage index following ginger or Cornelian cherry (COF) intervention in ovariectomized (OVX) rats under chronic mild stress (CMS).

	Sham-No	Sham-CMS	OVX-No	OVX-CMS	OVX-CMS-G	OVX-CMS-COF
Serum 17β-estradiol (pg/mL)	6.42 ± 0.56 ^a^	5.39 ± 0.75 ^b^	1.42 ± 0.13 ^d^	1.35 ± 0.16 ^d,^***^+##^	1.72 ± 0.12 ^c^	1.79 ± 0.19 ^c^
Uterine mass (g)	0.84 ± 0.06 ^a^	0.84 ± 0.02 ^a^	0.16 ± 0.02 ^c^	0.16 ± 0.01 ^c,^***	0.20 ± 0.01 ^b^	0.21 ± 0.01 ^b^
Final BW (g)	264 ± 10.2 ^b^	243 ± 8.2 ^c^	286 ± 12.1 ^a^	261 ± 12.7 ^b,^**^++^	251 ± 15.1 ^b^	259 ± 12.6 ^b^
Weight gain (g/5 weeks)	103 ± 6.55 ^b^	88.5 ± 6.18 ^c^	130 ± 6.52 ^a^	101 ± 6.12 ^b,^**^++^	95.6 ± 6.01 ^b^	99.3 ± 4.97 ^b^
Food intake (g/day)	17.2 ± 1.39 ^b^	15.5 ± 1.11 ^c^	19.8 ± 1.52 ^a^	16.7 ± 1.13 ^b,^*^+^	16.3 ± 1.49 ^bc^	16.5 ± 1.23 ^bc^
Uterine fat (g)	3.49 ± 0.30 ^b^	2.64 ± 0.54 ^c^	7.27 ± 0.44 ^a^	3.07 ± 0.32 ^c,^***^++##^	3.11 ± 0.23 ^bc^	3.20 ± 0.39 ^bc^
Retroperitoneal fat (g)	2.35 ± 0.36 ^b^	2.0 ± 0.20 ^bc^	3.85 ± 0.56 ^a^	2.52 ± 0.26 ^b,^**^++##^	1.79 ± 0.20 ^c^	2.41 ± 0.51 ^b^
Visceral fat (BW %)	2.22 ± 0.20 ^b^	1.35 ± 0.12 ^d^	3.69 ± 0.25 ^a^	2.04 ± 0.16 ^b,^**^++#^	1.75 ± 0.10 ^c^	1.94 ± 0.24 ^bc^
Fasting blood glucose (mg/dL)	98.1 ± 6.11 ^c^	104 ± 4.06 ^bc^	107 ± 3.03 ^b^	120 ± 4.09 ^a,^*^+#^	103 ± 5.73 ^bc^	102 ± 4.45 ^bc^
Serum insulin (ng/mL)	0.52 ± 0.02 ^c^	0.52 ± 0.01 ^c^	0.58 ± 0.01 ^a^	0.60 ± 0.02 ^a,^*^#^	0.55 ± 0.01 ^b^	0.54 ± 0.02 ^b^
HOMA-IR (µU/mL)	6.80 ± 0.47 ^c^	7.21 ± 0.49 ^bc^	8.28 ± 0.24 ^b^	9.60 ± 0.48 ^a,^***^+##^	7.54 ± 045 ^b^	7.34 ± 0.53 ^bc^
Serum AST (U/L)	24.4 ± 1.43 ^c^	25.9 ± 0.63 ^bc^	26.4 ± 1.33 ^b^	30.9 ± 1.15 ^a,^*^+##^	18.8 ± 1.15 ^d^	20.5 ± 1.56 ^d^
Serum ALT (U/L)	19.2 ± 2.36 ^b^	18.3 ± 2.27 ^b^	21.8 ± 2.02 ^a^	24.9 ± 3.33 ^a,^**^#^	17.7 ± 3.77 ^b^	16.5 ± 2.22 ^b^

Values represent means ± standard deviation (*n* = 10). Six experimental groups were included: sham-operated without CMS (Sham-No), sham-operated with CMS (Sham-CMS), OVX without CMS (OVX-No), OVX with CMS (OVX-CMS), OVX + CMS treated with ginger water extract (OVX-CMS-G), and OVX + CMS treated with Cornelian cherry water extract (OVX-CMS-COF). BW, body weight; HOMA-IR, homeostatic model assessment for insulin resistance; AST, aspartate aminotransferase; ALT, alanine aminotransferase. * Significant main effect of OVX in two-way ANOVA at *p* < 0.05, ** at *p* < 0.01, and *** at *p* < 0.001. ^+^ Significant main effect of CMS in two-way ANOVA at *p* < 0.05 and ^++^ at *p* < 0.01. ^#^ Significant interaction between OVX and CMS in two-way ANOVA at *p* < 0.05 and ^##^ at *p* < 0.01. ^a,b,c,d^ Different superscript letters indicate significant differences between all six groups in the Tukey post hoc test at *p* < 0.05.

**Table 3:** Biochemical and neurochemical parameters in serum and hippocampal tissue following ginger or Cornelian cherry (COF) intervention in ovariectomized (OVX) rats under chronic mild stress (CMS).

	Sham-No	Sham-CMS	OVX-No	OVX-CMS	OVX-CMS-G	OVX-CMS-COF
Serum						
TG (mg/dL)	80.3 ± 4.55 ^bc^	84.7 ± 4.69 ^b^	84.1 ± 3.58 ^b^	94.6 ± 5 ^a,^*^+#^	77.8 ± 4.44 ^c^	75.2 ± 5.41 ^c^
Total cholesterol (mg/dL)	130 ± 9.42 ^c^	128 ± 9.32 ^c^	147 ± 12.2 ^b^	166 ± 8.23 ^a,^**^++##^	128 ± 5.32 ^c^	147 ± 14.1 ^b^
HDL (mg/dL)	33.7 ± 2.64 ^a^	29.9 ± 3.11 ^b^	26 ± 2.47 ^c^	22.3 ± 1.56 ^d,^**^++^	27 ± 1.18 ^c^	29.1 ± 2.15 ^b^
TNF-α (ng/mL)	85.6 ± 7.21 ^d^	124 ± 10.2 ^c^	138 ± 11.6 ^b^	162 ± 13.8 ^a,^**^++^	136 ± 11.8 ^b^	131 ± 11.2 ^b^
IL-1β (ng/mL)	68.7 ± 7.21 ^c^	85.6 ± 8.14 ^b^	82.6 ± 7.91 ^b^	102 ± 10.8 ^a,^**^++^	89.6 ± 7.99 ^b^	87.6 ± 8.36 ^b^
Corticosterone (ng/mL)	78 ± 7.02 ^d^	103.5 ± 6.1 ^b^	94.2 ± 2.7 ^c^	118.7 ± 3.83 ^a,^**^+++^	103.2 ± 5.01 ^b^	104.2 ± 4.76 ^b^
ACTH (ng/mL)	3.23 ± 0.42 ^d^	6.35 ± 0.73 ^b^	4.93 ± 0.56 ^c^	8.52 ± 0.94 ^a,^***^+++^	6.23 ± 0.73 ^b^	6.31 ± 0.76 ^b^
Hippocampus						
Serotonin (ng/mg protein)	15.4 ± 2.05 ^a^	8.26 ± 0.71 ^b^	9.24 ± 1.02 ^b^	4.48 ± 0.29 ^d,^***^+++^	7.58 ± 1.02 ^c^	7.43 ± 0.94 ^c^
Dopamine (pg/mg protein)	7.81 ± 0.97 ^a^	5.83 ± 0.74 ^b^	6.48 ± 0.71 ^b^	4.54 ± 0.67 ^c,^**^++^	6.61 ± 0.42 ^b^	6.58 ± 0.53 ^b^
MDA (nmol/mg protein)	1.62 ± 0.24 ^c^	2.43 ± 0.28 ^b^	2.35 ± 0.28 ^b^	3.52 ± 0.32 ^a,^**^++^	2.16 ± 0.38 ^b^	2.39 ± 0.37 ^b^
SOD (U/mg protein)	54.3 ± 6.5 ^a^	47.9 ± 4.22 ^b^	46.5 ± 4.34 ^b^	40.4 ± 4.37 ^c,^**^+^	48.5 ± 4.31 ^b^	47.4 ± 4.42 ^b^

Sham-operated without CMS (Sham-No), sham-operated with CMS (Sham-CMS), OVX without CMS (OVX-No), OVX with CMS (OVX-CMS), OVX + CMS + ginger water extract (OVX-CMS-G), and OVX + CMS + Cornelian cherry water extract (OVX-CMS-COF). TG, triglyceride; HDL, high-density lipoprotein; TNF-α, tumor necrosis factor-α; IL-1β, interleukin-1β; ACTH, adrenocorticotropic hormone; MDA, malondialdehyde; SOD, superoxide dismutase. Values represented means ± standard deviation (*n* = 10). * Significant main effect of OVX in two-way ANOVA at *p* < 0.05, ** at *p* < 0.01, and *** at *p* < 0.001. ^+^ Significant main effect of CMS in two-way ANOVA at *p* < 0.05, ^++^ at *p* < 0.01, and, ^+++^ at *p* < 0.001. ^#^ Significant interaction between OVX and CMS in two-way ANOVA at *p* < 0.05 and ^##^ at *p* < 0.01. ^a,b,c,d^ Different superscript letters indicate significant differences between all six groups in the Tukey post hoc test at *p* < 0.05.

## Data Availability

Data is contained within the article or Supplementary Material.

## Supplementary Materials

The following supporting information can be downloaded at https://www.mdpi.com/article/10.3390/ijms26104829/s1.

## References

[1] Albert K.M., Newhouse P.A. (2019). Estrogen, Stress, and Depression: Cognitive and Biological Interactions. Annu. Rev. Clin. Psychol..

[2] Knezevic E., Nenic K., Milanovic V., Knezevic N.N. (2023). The Role of Cortisol in Chronic Stress, Neurodegenerative Diseases, and Psychological Disorders. Cells.

[3] Bucklin M.A., Gehrke E.C., Westrick J.C., Gottlieb M., Martin J.T. (2024). Depression predicts decreased lumbar bone mineral density: A scoping review of chronic psychological stress and spinal tissue pathology. Osteoarthr. Cartil. Open.

[4] Yang F., Liu Y., Chen S., Dai Z., Yang D., Gao D., Shao J., Wang Y., Wang T., Zhang Z. (2020). A GABAergic neural circuit in the ventromedial hypothalamus mediates chronic stress-induced bone loss. J. Clin. Investig..

[5] Sun Q., Li G., Zhao F., Dong M., Xie W., Liu Q., Yang W., Cui R. (2024). Role of estrogen in treatment of female depression. Aging.

[6] Tongta S., Daendee S., Kalandakanond-Thongsong S. (2023). Anxiety-like behavior and GABAergic system in ovariectomized rats exposed to chronic mild stress. Physiol. Behav..

[7] Hou H., Adzika G.K., Wu Q., Ma T., Ma Y., Geng J., Shi M., Fu L., Rizvi R., Gong Z. (2021). Estrogen Attenuates Chronic Stress-Induced Cardiomyopathy by Adaptively Regulating Macrophage Polarizations via β_2_-Adrenergic Receptor Modulation. Front. Cell Dev. Biol..

[8] Ayustaningwarno F., Anjani G., Ayu A.M., Fogliano V. (2024). A critical review of Ginger’s (*Zingiber officinale*) antioxidant, anti-inflammatory, and immunomodulatory activities. Front. Nutr..

[9] Gao X., Liu Y., An Z., Ni J. (2021). Active Components and Pharmacological Effects of *Cornus officinalis*: Literature Review. Front. Pharmacol..

[10] Tian W., Zhao J., Lee J.H., Akanda M.R., Cho J.H., Kim S.K., Choi Y.J., Park B.Y. (2019). Neuroprotective Effects of *Cornus officinalis* on Stress-Induced Hippocampal Deficits in Rats and H_2_O_2_-Induced Neurotoxicity in SH-SY5Y Neuroblastoma Cells. Antioxidants.

[11] Zhang Y., Yuan P.-P., Li P.-Y., Zheng Y.-J., Li S.-F., Zhao L.-R., Ma Q.Y., Cheng J.L., Ma J.S., Feng W.S. (2025). Investigating the possible mechanism of *Cornus officinalis* in the therapy of ischemic stroke by UHPLC-Q-TOF-MS, network pharmacology, molecular docking, and experimental verification. J. Ethnopharmacol..

[12] Bakusic J., Vrieze E., Ghosh M., Bekaert B., Claes S., Godderis L. (2020). Increased methylation of NR3C1 and SLC6A4 is associated with blunted cortisol reactivity to stress in major depression. Neurobiol. Stress.

[13] Xiao Z., Liu H. (2024). The estrogen receptor and metabolism. Women’s Health.

[14] Qiu W., Cai X., Zheng C., Qiu S., Ke H., Huang Y. (2021). Update on the Relationship Between Depression and Neuroendocrine Metabolism. Front. Neurosci..

[15] Jiang Y., Zou D., Li Y., Gu S., Dong J., Ma X., Xu S., Wang F., Huang J.H. (2022). Monoamine Neurotransmitters Control Basic Emotions and Affect Major Depressive Disorders. Pharmaceuticals.

[16] Razak A.M., Tan J.K., Mohd Said M., Makpol S. (2023). Modulating Effects of Zingiberaceae Phenolic Compounds on Neurotrophic Factors and Their Potential as Neuroprotectants in Brain Disorders and Age-Associated Neurodegenerative Disorders: A Review. Nutrients.

[17] Giannini A., Caretto M., Genazzani A.R., Simoncini T. (2021). Neuroendocrine Changes during Menopausal Transition. Endocrines.

[18] Turek J., Gąsior Ł. (2023). Estrogen fluctuations during the menopausal transition are a risk factor for depressive disorders. Pharmacol. Rep..

[19] Zhang Z., He Z., Pan J., Yuan M., Lang Y., Wei X., Zhang C. (2024). The interaction of BDNF with estrogen in the development of hypertension and obesity, particularly during menopause. Front. Endocrinol..

[20] Corrigan M., O’Rourke A.M., Moran B., Fletcher J.M., Harkin A. (2023). Inflammation in the pathogenesis of depression: A disorder of neuroimmune origin. Neuronal Signal..

[21] Tongta S., Daendee S., Kalandakanond-Thongsong S. (2022). Effects of estrogen receptor β or G protein-coupled receptor 30 activation on anxiety-like behaviors in relation to GABAergic transmission in stress-ovariectomized rats. Neurosci. Lett..

[22] Khaleghi M., Rajizadeh M.A., Bashiri H., Kohlmeier K.A., Mohammadi F., Khaksari M., Shabani M. (2021). Estrogen attenuates physical and psychological stress-induced cognitive impairments in ovariectomized rats. Brain Behav..

[23] Brann D.W., Lu Y., Wang J., Zhang Q., Thakkar R., Sareddy G.R., Pratap U.P., Tekmal R.R., Vadlamudi R.K. (2022). Brain-derived estrogen and neural function. Neurosci. Biobehav. Rev..

[24] Green M.R., Marcolin M.L., McCormick C.M. (2018). The effects of ovarian hormones on stressor-induced hormonal responses, glucocorticoid receptor expression and translocation, and genes related to receptor signaling in adult female rats. Stress.

[25] Bhattacharya A., Chakraborty M., Chanda A., Alqahtani T., Kumer A., Dhara B., Chattopadhyay M. (2024). Neuroendocrine and cellular mechanisms in stress resilience: From hormonal influence in the CNS to mitochondrial dysfunction and oxidative stress. J. Cell. Mol. Med..

[26] Hei M., Chen P., Wang S., Li X., Xu M., Zhu X., Wang Y., Duan J., Huang Y., Zhao S. (2019). Effects of chronic mild stress induced depression on synaptic plasticity in mouse hippocampus. Behav. Brain Res..

[27] Chaurasiya N.D., Leon F., Muhammad I., Tekwani B.L. (2022). Natural Products Inhibitors of Monoamine Oxidases-Potential New Drug Leads for Neuroprotection, Neurological Disorders, and Neuroblastoma. Molecules.

[28] Shaikh A., Ahmad F., Teoh S.L., Kumar J., Yahaya M.F. (2023). Targeting dopamine transporter to ameliorate cognitive deficits in Alzheimer’s disease. Front. Cell. Neurosci..

[29] Boyle C.C., Bower J.E., Eisenberger N.I., Irwin M.R. (2023). Stress to inflammation and anhedonia: Mechanistic insights from preclinical and clinical models. Neurosci. Biobehav. Rev..

[30] Ruggiero R.N., Rossignoli M.T., Marques D.B., de Sousa B.M., Romcy-Pereira R.N., Lopes-Aguiar C., Leite J.P. (2021). Neuromodulation of Hippocampal-Prefrontal Cortical Synaptic Plasticity and Functional Connectivity: Implications for Neuropsychiatric Disorders. Front. Cell. Neurosci..

[31] Rong J., Wang Y., Liu N., Shen L., Ma Q., Wang M., Han B. (2024). Chronic stress induces insulin resistance and enhances cognitive impairment in AD. Brain Res. Bull..

[32] Park S., Hong S.M., Ahn I.L., Kim D.S., Kim S.H. (2010). Estrogen replacement reverses olanzapine-induced weight gain and hepatic insulin resistance in ovariectomized diabetic rats. Neuropsychobiology.

[33] Beaupere C., Liboz A., Fève B., Blondeau B., Guillemain G. (2021). Molecular Mechanisms of Glucocorticoid-Induced Insulin Resistance. Int. J. Mol. Sci..

[34] Daily J.W., Zhang X., Kim D.S., Park S. (2015). Efficacy of Ginger for Alleviating the Symptoms of Primary Dysmenorrhea: A Systematic Review and Meta-analysis of Randomized Clinical Trials. Pain Med..

[35] Cozma D., Siatra P., Oikonomakos I., Kalra D., Friedrich U.A., Dahl A., Bornstein S.R., Zeissig S., Andoniadou C.L., Steenblock C. (2023). SAT272 Insulin Signaling in Hyperactivation of the HPA Axis in Metabolic Diseases. J. Endocr. Soc..

[36] Kumar S., Kumar B.H., Nayak R., Pandey S., Kumar N., Pai K.S.R. (2025). Computational screening and molecular dynamics of natural compounds targeting the SH2 domain of STAT3: A multitarget approach using network pharmacology. Mol. Divers..

[37] Geha R.M., Chen K., Wouters J., Ooms F., Shih J.C. (2002). Analysis of conserved active site residues in monoamine oxidase A and B and their three-dimensional molecular modeling. J. Biol. Chem..

[38] Kim K., Che T., Panova O., DiBerto J.F., Lyu J., Krumm B.E., Wacker D., Robertson M.J., Seven A.B., Nichols D.E. (2020). Structure of a Hallucinogen-Activated Gq-Coupled 5-HT_2A_ Serotonin Receptor. Cell.

[39] Shi Y., Cao S., Ni D., Fan J., Lu S., Xue M. (2022). The Role of Conformational Dynamics and Allostery in the Control of Distinct Efficacies of Agonists to the Glucocorticoid Receptor. Front. Mol. Biosci..

[40] Zhu J., Chen H., Song Z., Wang X., Sun Z. (2018). Effects of Ginger (*Zingiber officinale* Roscoe) on Type 2 Diabetes Mellitus and Components of the Metabolic Syndrome: A Systematic Review and Meta-Analysis of Randomized Controlled Trials. Evid. Based Complement. Altern. Med..

[41] Simon A., Darcsi A., Kéry Á., Riethmüller E. (2020). Blood-brain barrier permeability study of ginger constituents. J. Pharm. Biomed. Anal..

[42] Andrei C., Zanfirescu A., Nițulescu G.M., Negreș S. (2022). Understanding the Molecular Mechanisms Underlying the Analgesic Effect of Ginger. Nutraceuticals.

[43] Yang H.J., Zhang T., Kim M.J., Hur H.J., Wu X., Jang D.J., Park S. (2024). Efficacy and Mechanism of Schisandra chinensis Fructus Water Extract in Alzheimer’s Disease: Insights from Network Pharmacology and Validation in an Amyloid-β Infused Animal Model. Nutrients.

[44] Zhou J., Kim Y.K., Li C., Park S. (2025). Natural compounds for Alzheimer’s prevention and treatment: Integrating SELFormer-based computational screening with experimental validation. Comput. Biol. Med..

[45] Liu M., Park S. (2024). The Role of PNPLA3_rs738409 Gene Variant, Lifestyle Factors, and Bioactive Compounds in Nonalcoholic Fatty Liver Disease: A Population-Based and Molecular Approach towards Healthy Nutrition. Nutrients.

[46] Pavlova I.V., Broshevitskaya N.D., Zaichenko M.I., Grigoryan G.A. (2022). Effects of Ovariectomy on Anxious-Depressive Behavior in Female Rats in Normal Conditions and after Early Proinflammatory Stress. Neurosci. Behav. Physiol..

[47] Santos J.M., Deshmukh H., Elmassry M.M., Yakhnitsa V., Ji G., Kiritoshi T., Presto P., Antenucci N., Liu X., Neugebauer V. (2024). Beneficial Effects of Ginger Root Extract on Pain Behaviors, Inflammation, and Mitochondrial Function in the Colon and Different Brain Regions of Male and Female Neuropathic Rats: A Gut-Brain Axis Study. Nutrients.

[48] Park E., Lim E., Yeo S., Yong Y., Yang J., Jeong S.Y. (2020). Anti-Menopausal Effects of *Cornus officinalis* and *Ribes fasciculatum* Extract In Vitro and In Vivo. Nutrients.

[49] Ju C.G., Zhu L., Wang W., Gao H., Xu Y.B., Jia T.Z. (2022). *Cornus officinalis* prior and post-processing: Regulatory effects on intestinal flora of diabetic nephropathy rats. Front. Pharmacol..

[50] Seo-Ah S., Sung-Chul K., Bo-Ram J., Min-jeong K., Sujung Y., In-hwa P., Hyo-Jin A. (2021). A 13-Week Repeated Oral Dose Toxicity Test and a 4-Week Recovery Test of Standardized *Cornus officinalis* and *Psoralea corylifolia* L. in Sprague-Dawley Rats. Korea J. Herbol..

[51] Willner P. (2017). The chronic mild stress (CMS) model of depression: History, evaluation and usage. Neurobiol. Stress.

[52] Zhang T., Kim M.J., Kim M.J., Wu X., Yang H.J., Yuan H., Huang S., Yoon S.M., Kim K.N., Park S. (2022). Long-Term Effect of Porcine Brain Enzyme Hydrolysate Intake on Scopolamine-Induced Memory Impairment in Rats. Int. J. Mol. Sci..

[53] Markov D.D. (2022). Sucrose Preference Test as a Measure of Anhedonic Behavior in a Chronic Unpredictable Mild Stress Model of Depression: Outstanding Issues. Brain Sci..

[54] Cheah M., Fawcett J.W., Andrews M.R. (2017). Assessment of Thermal Pain Sensation in Rats and Mice Using the Hargreaves Test. Bio Protoc..

[55] Park S., Kang S., Kim D.S. (2020). Severe calcium deficiency increased visceral fat accumulation, down-regulating genes associated with fat oxidation, and increased insulin resistance while elevating serum parathyroid hormone in estrogen-deficient rats. Nutr. Res..

[56] Peinnequin A., Mouret C., Birot O., Alonso A., Mathieu J., Clarençon D., Agay D., Chancerelle Y., Multon E. (2004). Rat pro-inflammatory cytokine and cytokine-related mRNA quantification by real-time polymerase chain reaction using SYBR green. BMC Immunol..

[57] Livak K.J., Schmittgen T.D. (2001). Analysis of relative gene expression data using real-time quantitative PCR and the 2^−ΔΔ*C*^_T_ Method. Methods.

