# Supplementary material for: Network Pharmacology-Guided Evaluation of Ginger and Cornelian Cherry Extracts Against Depression and Metabolic Dysfunction in Estrogen-Deficient Chronic Stressed Rats
Source: Int J Mol Sci. 2025 May 18;26(10):4829. doi: 10.3390/ijms26104829 (PMC12112620; doi:10.3390/ijms26104829)
Supplement: Supplementary file 1 [file ijms-26-04829-s001.zip › ijms-3552662-supplementary.pdf]

Table S1. Oral availability (OB) and drug likeness (DL) in components in from *Zingiber officinale* Roscoe and *Cornus officinalis* Sieb. Et Zucc. fruit

Latin name	Mol ID	Mol name	OB(%)	DL
Ginger *Zingiber officinale* Roscoe	MOL002467	6-gingerol	35.63	0.16
	MOL002495	6-shogaol	31	0.15
	MOL000358	beta-sitosterol	36.91	0.75
	MOL006129	6-methylgingediacetate2	48.73	0.32
	MOL000449	Stigmasterol	43.82	0.75
	MOL001771	Poriferast-5-en-3beta-ol	36.91	0.75
	MOL003358	Euxanthone	92.98	0.16
	MOL008698	Dihydrocapsaicin	47.07	0.19
Shanzhuyu (山茱萸) *Cornus officinalis* Sieb. Et Zucc.	MOL001398	Methyllinolenate	46.15	0.17
	MOL001494	Mandenol	41.99	0.19
	MOL001495	Ethyl linolenate	46.1	0.19
	MOL001771	poriferast-5-en-3beta-ol	36.91	0.75
	MOL001889	Methyl linolelaidate	41.93	0.16
	MOL002879	Diop	43.59	0.39
	MOL002883	Ethyl oleate (NF)	32.39	0.19
	MOL003137	Leucanthoside	32.11	0.78
	MOL000358	beta-sitosterol	36.91	0.75
	MOL000359	Sitosterol	36.91	0.75
	MOL000449	Stigmasterol	43.82	0.75
	MOL005360	Malkangunin	57.71	0.62
	MOL005478	11,14-Octadecadienoic acid, methyl ester	41.93	0.16
	MOL005481	2,6,10,14,18-pentamethylicosa-2,6,10,14,18-pentaene	33.4	0.24
	MOL005485	3-dibenzofuransulfonic acid	74.42	0.15
	MOL005486	3,4-Dehydrolycopen-16-al	46.64	0.49
	MOL005489	3,6-Digalloylglucose	31.41	0.66
	MOL005503	Cornudentanone	39.66	0.32
	MOL005530	Hydroxygenkwanin	36.46	0.27
	MOL005531	Telocinobufagin	69.99	0.79
	MOL008457	Tetrahydroalstonine	32.41	0.81
	MOL000554	Gallic acid-3-O-(6'-O-galloyl)-Glucoside	30.25	0.67
	MOL005552	Gemin D	68.83	0.56
	MOL005557	lanosta-8,24-dien-3-ol,3-acetate	44.29	0.82

Table S2. The binding energy between natural compounds and proteins related to chronic stress and estrogen deficiency (unit: kcal/mol)

Protein	6-gingerol	6-shogaol	Cornudentanone	Hydroxygenkwanin	Telocinobufagin	Tetrahydroalstonine
MAOA	-5.7	-6.9	-6	-8.4	-7.7	-6.7
HTR2A	-5.6	-5.8	-5.3	-8.5	-7.6	-6.7
SLC6A3	-5.4	-6	-5.4	-6.9	-7.4	-7.2
SLC6A4	-5.8	-5.9	-6.1	-7.1	-7.9	-7.5
NR3C1	-4.8	-4.9	-5.3	-6.4	-8.4	-6.1

Table S3. Chronic mild stress protocol

Week	Sunday	Monday	Tuesday	Wednesday	Thursday	Friday	Saturday
Week 1	WB (24h)	SI (9h) + OF (2h)	FR (24h) + CT (16h)	WB (24h)	SI (24h) + FST (5min)	CT (24h)	WC (9h)
Week 2	WB (24h)	CT (24h) + RS (3h) + OF (2h)	FR (24h) + CT (16h)	WB (24h) + WD (16h)	CT (24h) + CT (9h)	SI (24h) + FS (16h)	WC (9h)
Week 3	WB (24h)	ES (9h) + FD (16h)	WC (24h)	SI (24h) + RS (3h) + OF (2h)	CT (24h) + CT (9h)	CT (24h) + FST(5min)	WC (9h)
Week 4	WB (24h)	CT (24h) + RS (3h)	WC(24h) + WD(16h)	CT (24h) + CT (16h)	SI (24h) + FST (5min)	CT (24h)	WC (9h)
Week 5	WB (24h)	ES (9h) + CT (24h)	CT (24h) + FR (16h)	WC (24h)	SI (24h) + RS (3h) + OF(2h)	CT (24h)	WC (9h)

WB: Wet Bedding - bedding material saturated with water; WD: Water Deprivation - restricted access to water; FR/FD: Food Restriction/Food Deprivation - limited access to food; WC: Wet Cage - cage floor covered with water; ES: Electric Shock Warning Box - exposure to box with warning signals; CT: Cold Temperature - exposure to reduced ambient temperature; SI: Social Isolation - individual housing; RS: Restraint Stress - physical restraint; OF: Open Field Test - behavioral assessment in open arena; FST/FS: Forced Swim Test/Forced Swimming - swimming in water tank

Supplementary Figure S1. Chromatogram of *Zingiber officinale* Roscoe (A) and *Cornus officinalis* Siebold & Zucc. Fruit water extracts (B)

A.

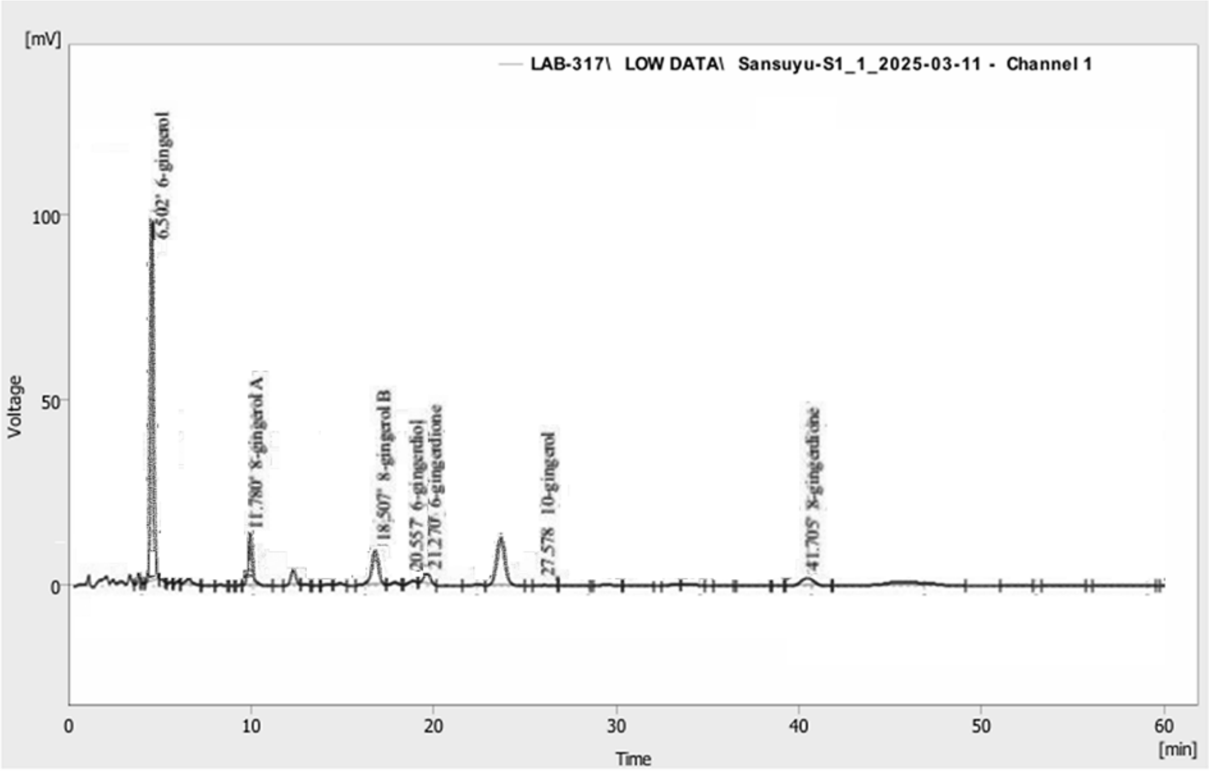

B.

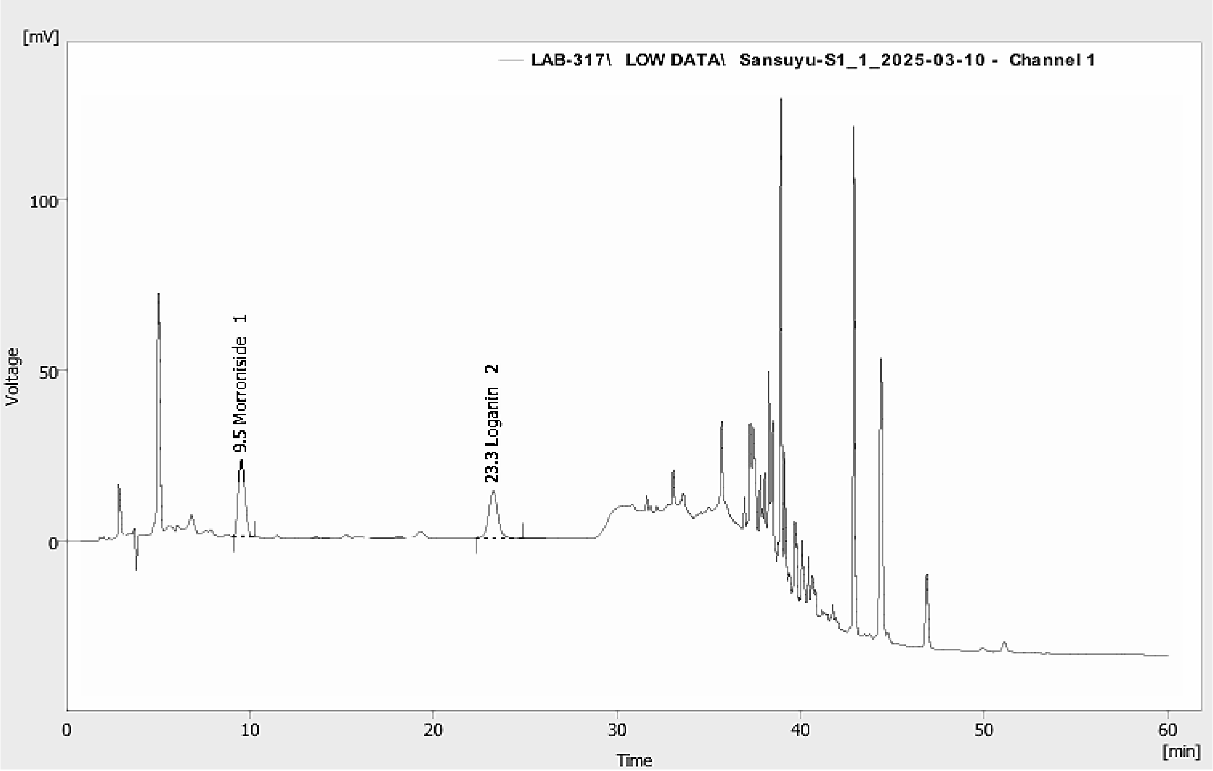

Figure S2. Molecular docking between proteins and selected natural compounds.

A. MAOA (PDB: 2BXR) and Clorgyline (binding energy = -5.0)

B. HTR2A (PDB: 6A93) and risperidone (binding energy = -8.5)

C. NR3C1 (PDB: 1NHZ) and 11-(4-dimethylamino-phenyl)-17-hydroxy-13-methyl-17-prop-1-ynyl-1,2,6,7,8,11,12,13,14,15,16,17-dodecahydro cyclopent[A]phenanthrene-3-one (binding energy = -7.4).

S2A

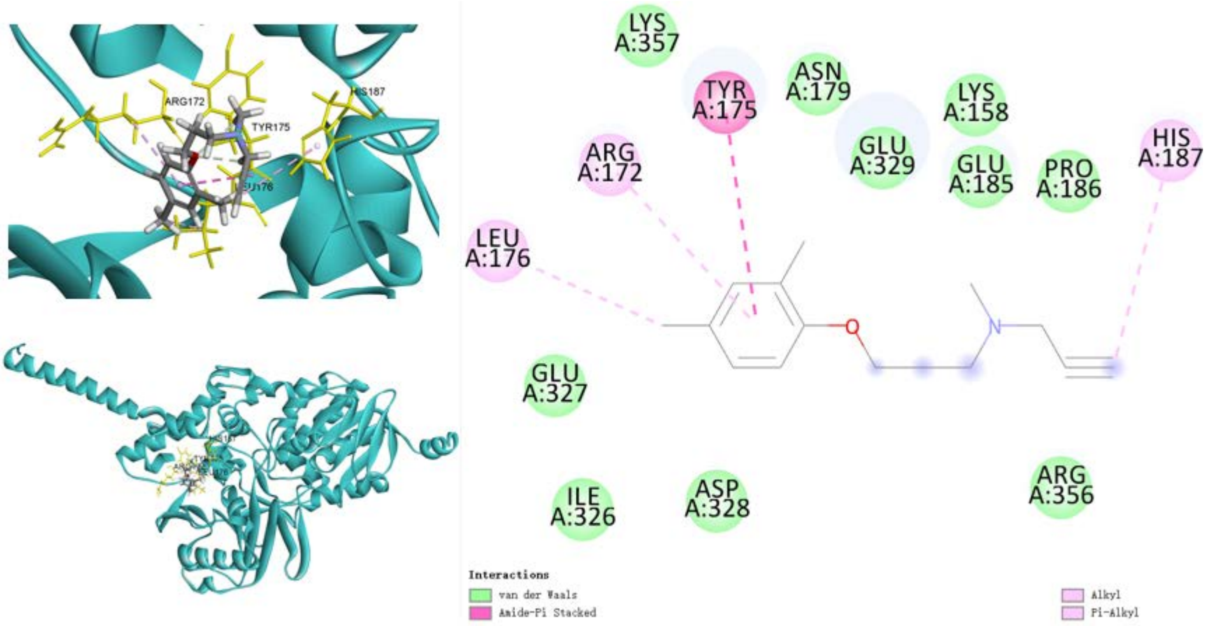

S2B

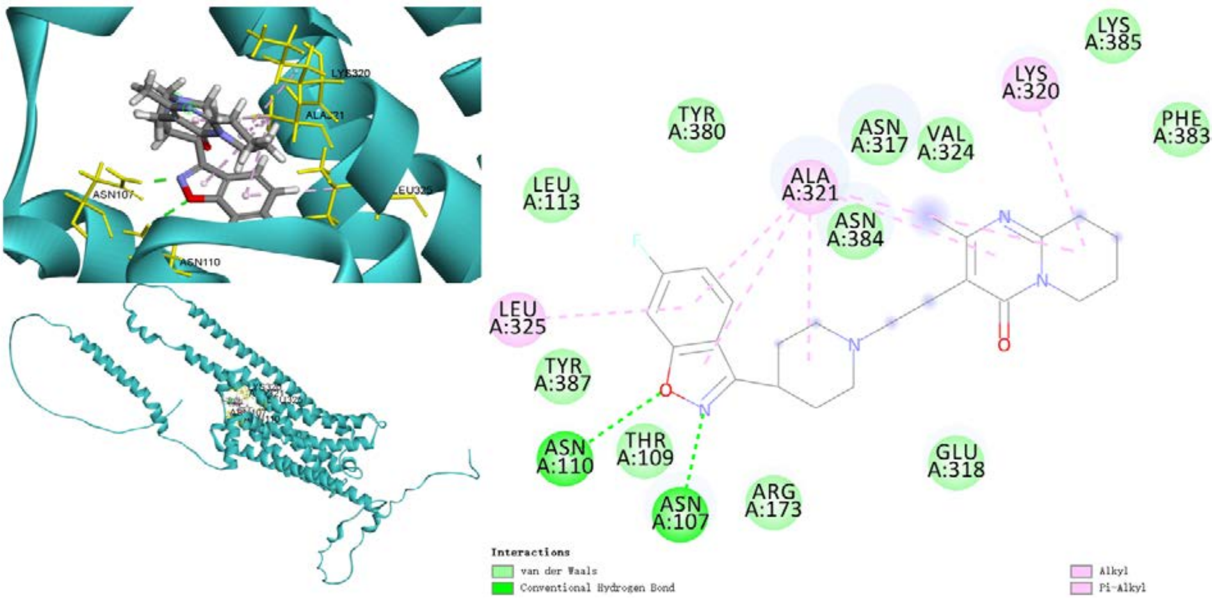

S2C

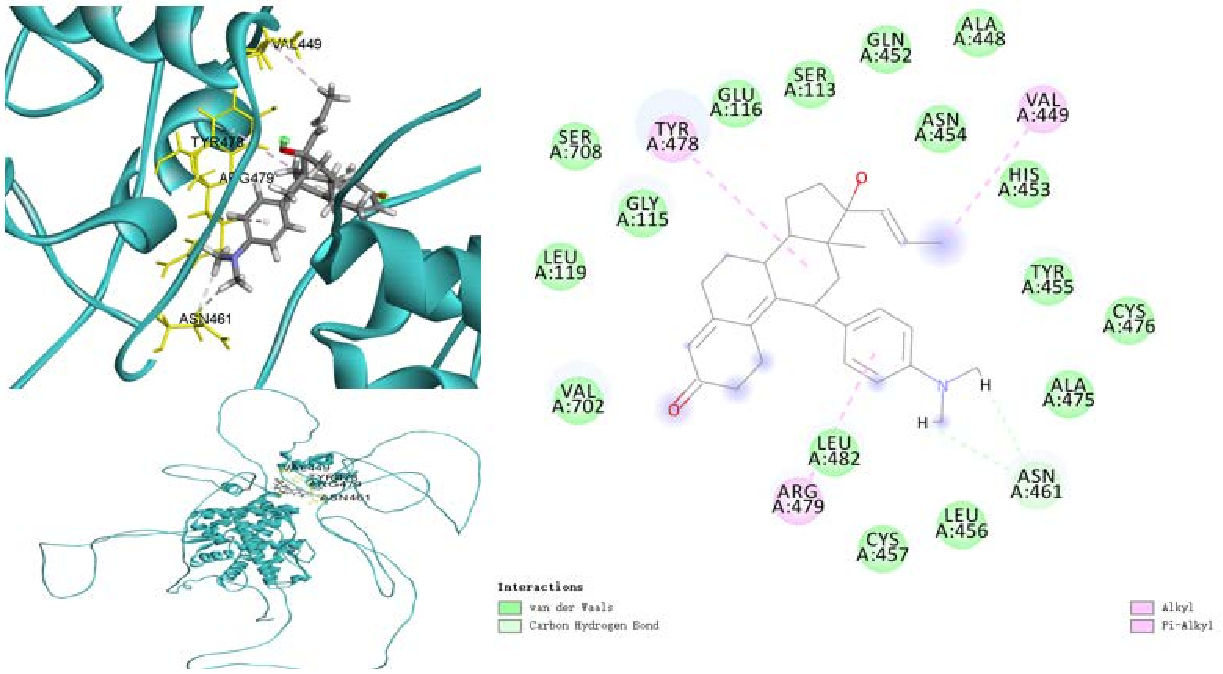
VAL449
TYR478
ARG479
ASN461
ALA A:448
GLN A:452
SER A:113
GLU A:116
TYR A:478
SER A:708
ASN A:454
VAL A:449
HIS A:453
GLY A:115
LEU A:119
TYR A:455
CYS A:476
VAL A:702
ALA A:475
LEU A:482
ASN A:461
ARG A:479
LEU A:456
CYS A:457
Interactions
van der Waals
Carbon Hydrogen Bond
Alkyl
Pi-Alkyl
